# Preliminary construction and validation of a prognostic prediction model for cervical cancer based on tumor mechanics-related genes

**DOI:** 10.3389/fonc.2026.1841456

**Published:** 2026-06-03

**Authors:** Lu Zhang, Haonan Fu, Yali Feng, Jianxin Tian, Xue Gao, Yuhong Shang

**Affiliations:** 1 Department of Obstetrics and Gynecology, The First Affiliated Hospital of Dalian Medical University, Dalian, Liaoning, China; 2 Department of Pathology, The First Affiliated Hospital of Dalian Medical University, Dalian, Liaoning, China

**Keywords:** cervical cancer, MMP1, prognostic model, strain elastography, tumor mechanics, tumor stiffness

## Abstract

**Objective:**

Cervical cancer (CC) is the fourth most prevalent malignancy among women. The present study employed bioinformatics analyses to identify tumor mechanics-related genes (TMRGs), establish a prognostic model, and investigate the association of tumor stiffness with pivotal genes utilizing clinical samples.

**Methods:**

mRNA data from the Genotype-Tissue Expression project (GTEx), The Cancer Genome Atlas (TCGA), and the Gene Expression Omnibus (GEO) were analyzed to identify tumor mechanics-related differentially expressed genes. A prognostic model was constructed via least absolute shrinkage and selection operator (LASSO) regression and validated in theGSE44001cohort. In the clinical component, individuals with CC were enrolled; the preoperative strain ratio (SR), reflecting tumor stiffness, was evaluated by strain elastography, and the expression of matrix metalloproteinase-1 (MMP1) in tumor tissues was evaluated using immunohistochemistry (IHC).

**Results:**

A prognostic model was constructed using seven key genes: MMP1, DES, ARSJ, NT5E, P4HA3, CLMP, and SMARCA1. This model efficiently stratified individuals into high- and low-risk subgroups. The two groups exhibited distinct gene mutation landscapes, varying degrees of immune cell infiltration, and differential responses to chemotherapy. Spearman correlation analysis indicated a moderate positive link between MMP1 IHC scores and SR values (r=0.418, P = 0.012).Compared to the low-expression group, individuals with high MMP1expressionexhibited significantly elevated SR values(P<0.05).

**Conclusions:**

The TMRG-based prognostic model demonstrated notable discriminative capacity. Clinical validation revealed a preliminary association between tumor stiffness and MMP1 expression in CC tissues, offering a new perspective for risk stratification and clinical evaluation in this malignancy.

## 1 Introduction

Cervical cancer (CC) ranks as the fourth most frequent malignancy among women, with an estimated 600,000 new cases and 340,000 deaths annually (1). Despite advances in screening programs that have reduced CC incidence, survival rates remain low for individuals diagnosed at advanced stages (2). Thus, identification of effective biomarkers and prognostic signatures to improve CC outcomes is a critical unmet need.

The role of tumor biology has gained increasing attention in the context of tumor development. Deviations from the mechanical properties of normal tissues represent a hallmark of tumors, acting as significant determinants of initiation, progression, metastasis, and therapeutic response (1). Tumor stiffness, a representative mechanical parameter, serves as a central player in tumor pathophysiology. Clinical studies have illustrated elevated stiffness in solid malignancies, including pancreatic, breast, and colorectal cancers, compared to their normal counterparts (3, 4). This phenomenon relates to the mechanical imbalance within the tumor microenvironment (TME). Disruption of the dynamic equilibrium involving extracellular matrix (ECM) deposition and degradation, abnormalities in tumor cell density regulation due to aberrant tumor cell growth, and elevated interstitial fluid pressure resulting from lymphatic system disruption (5, 6) contribute to pathological increase in tumor stiffness.

The development of ultrasound elastography has provided tools to quantify tissue stiffness effectively. Since its introduction in 1991 (7), this technology has been widely adopted in the diagnosis of thyroid, breast, and liver diseases (8–10). More recently, its application has expanded to gynecology and obstetrics, where it holds promise for measuring cervical tissue stiffness, thus informing CC diagnosis and management. Published data support elevated lesion stiffness in CC compared to normal cervical tissue, benign cervical conditions (11), and premalignant lesions (12). Thus, tumor stiffness emerges as a potential biomarker for CC progression.

Prior investigation has identified 79 tumor mechanics-related genes (TMRGs), which are critical regulators of cell motility and ECM stiffness (13). Building on this knowledge, the current study leveraged The Cancer Genome Atlas (TCGA) data on cervical squamous cell carcinoma and adenocarcinoma (CESC) to isolate key TMRGs, build a prognostic model, and validate it within the Gene Expression Omnibus (GEO) datasets. The biological functions involved in the model and the characteristics of the tumor immune microenvironment were explored. Additionally, a cohort of CC individuals was enrolled in the clinical portion to evaluate lesion stiffness via preoperative strain elastography (SE) and correlate it with matrix metalloproteinase 1 (MMP1) expression levels in lesion tissues.

## 2 Methods and materials

### 2.1 Data acquisition

mRNA expression profiles and clinical data for TCGA-CESC subjects were retrieved from the University of California, Santa Cruz (UCSC) Xena platform (https://xena.ucsc.edu/). Transcriptomic profiles of non-cancerous cervical tissues were acquired from the Genotype-Tissue Expression (GTEx) project. The GSE44001 dataset, sourced from the GEO repository, was adopted for external validation. The TMRG set comprised 79 genes identified in the published literature [**Supplementary Table 1;** (13)].

### 2.2 Differential expression analysis and selection of TMRGs

Transcriptomic data (raw counts) from 291 CC samples and 3 normal tissue samples from the TCGA-CESC cohort, together with 88 normal cervical tissue samples from the GTEx database, were integrated for analysis. To ensure analytical robustness, low-expression genes were filtered using a stringent criterion: only genes with non-zero counts in at least 30% of samples were retained. Differential expression analysis was carried out employing the R package ‘DESeq2’ (v1.36.0). To account for possible batch effects arising from distinct data sources (TCGA and GTEx), a batch factor was incorporated as a covariate into the design formula of the generalized linear model: design = ~ batch + group. This approach performs statistical correction for batch effects directly on raw counts during model fitting, rather than relying on pre-normalized data. Differentially expressed genes (DEGs) between CC and normal tissues were identified using thresholds of |log_2_FC|≥0.585 and P<0.05.

According to the expression profiles of the 79 TMRGs, consensus clustering was implemented in TCGA-CESC tumor samples, yielding C1 and C2 subtypes. The ‘DESeq2’ package(v1.36.0) was then adopted to identify DEGs between these subtypes (|log2FC|≥1 and P<0.05), thereby capturing potential tumor mechanics-related differentially expressed genes (MEDEGs). Survival differences between C1 and C2 subtypes were evaluated via Kaplan-Meier (KM) analysis with log-rank testing.

The MEDEGs in CC were identified by intersecting two gene sets: (i) DEGs between CC and normal tissues, and (ii) previously reported MEDEGs.

### 2.3 Establishment and verification of a TMRG-based prognostic model

By integrating expression profiles of MEDEGs with survival information, univariate Cox regression was applied to identify genes associated with prognosis (P<0.05). Using the ‘glmnet’ (v4.1-4) and ‘survival’ (v3.3-1) packages, LASSO regression was performed with the TCGA-CESC cohort as the training set and the GSE44001 cohort as the validation set (detailed clinical characteristics and sequencing platforms for both cohorts are provided in **Supplementary Table 2**). Optimism correction via bootstrap resampling (1,000 iterations) was adopted to mitigate overfitting. Model stability was further evaluated through repeated construction using different random seeds and a 6:4 data-splitting strategy. The λ value yielding the minimum error was determined by cross-validation to select pivotal genes for constructing the prognostic model. The risk score was calculated as: Risk score=Σ (Gene expression level × Regression coefficient). Participants were dichotomized into low- and high-risk groups according to the median risk score. KM survival analysis was conducted with the ‘survival’ (v3.3-1) and ‘survminer’ (v0.4.9) packages. Model performance was assessed via time-dependent receiver operating characteristic (ROC) curves using the ‘timeROC’ (v0.4) package. The identical risk score formula was applied to the GSE44001 cohort for external validation.

### 2.4 Independent prognostic analysis and nomogram

Univariate and multivariate Cox regression models were employed to establish independent prognostic factors. Utilizing the ‘rms’(v6.3-0) and ‘survival’(v3.3-1)packages, a nomogram was constructed to integrate the risk score with independent clinical predictors (histological grade, age, stage, etc.) for estimating 1-, 2-, and 3-year survival probabilities. Model robustness was evaluated via calibration curves, ROC curves, and C-index calculation.

### 2.5 Mutational landscape analysis

Mutation data for TCGA-CESC subjects were acquired via the ‘TCGAbiolinks’ package(v2.24.3). The ‘maftools’ package(v2.12.0) was adopted to analyze differences in gene mutation profiles between the high- and low-risk groups.

### 2.6 Immune cell infiltration analysis

ICI scores for 22 distinct immune cell types were calculated for TCGA-CESC subjects utilizing the CIBERSORT algorithm as implemented in the ‘IOBR’ package(v0.99.9).

The differences of ICI between low- and high-risk groups were compared, followed by Spearman correlation analysis to evaluate the correlation between risk scores and infiltration abundance(P<0.05 indicating statistical significance).

### 2.7 Drug sensitivity analysis

Utilizing the ‘pRRophetic’ package(v0.5), the half-maximal inhibitory concentration (IC50) was estimated for anticancer agents across all CESC samples. Box plots were created to visualize differential drug sensitivity between the two risk groups. The intergroup differences were compared. A P-value of <0.001 was selected for screening. Additionally, Spearman correlation coefficients between the risk score and drug sensitivities were computed via the ‘cor.test’ function, utilizing thresholds of P<0.001 and |correlation coefficient|>0.3.

### 2.8 Single-gene pan-cancer analysis

The ‘TCGAplot’ package(v0.0.1) was adopted to analyze the expression profiles of key genes in both cancer and non-cancerous tissues across a range of cancers, as well as in paired cancer and adjacent normal tissues. Kyoto Encyclopedia of Genes and Genomes (KEGG) and Gene Ontology (GO) enrichment analyses were implemented for MMP1.

### 2.9 Single-gene expression validation and survival analysis

The expression levels of core genes in tumor and normal tissues within TCGA-CESC were compared. ROC curves were generated via the ‘pROC’ package(v1.18.0). Survival analysis was performed on each of the seven core genes within the TCGA-CESC dataset, grouped based on the optimal threshold, utilizing the ‘survival’(v3.3-1) and ‘survminer’ (v0.4.9) packages.

### 2.10 Clinical sample validation

This study was conducted in accordance with the Declaration of Helsinki and was approved by the Ethics Committee of The First Affiliated Hospital of Dalian Medical University (Approval No.: [PJ-KS-KY-2025-333]).

#### 2.10.1 Strain elastography

Subjects with suspected CC presenting to the hospital between July and December 2023, and between January and July 2025, were enrolled for SE evaluation. SE was performed utilizing a GE Voluson E10 ultrasound platform with a 5–9 MHz probe. After emptying their bladder, subjects were placed in the lithotomy position. The probe was gently positioned within the vaginal fornix. Two-dimensional ultrasound imaging was performed to assess lesion echogenicity, margins, size, shape, and location. The elastography mode was then activated, ensuring that the image center was positioned within the region of interest and that the sampling frame encompassed both the lesion and surrounding normal tissue. A light pressure was applied to the cervix at a frequency of 2–3 times per second. The image was frozen when the pressure was constant. The color distribution within the lesion was recorded. The region of interest was defined within the lesion, as well as an adjacent region of similar depth and size. Strain rates were measured in both regions. The strain ratio (SR) was automatically computed. These procedures were all carried out by a single, highly experienced gynecological ultrasonographer. All enrolled participants subsequently underwent surgery, which confirmed the diagnosis of CC in 35 cases. Inclusion criteria comprised: (i) individuals with clinical manifestations or routine ultrasound findings suspicious for CC; (ii) those who underwent ultrasound elastography at our institution preoperatively; (iii) those who received surgical treatment at our institution post-examination with available postoperative pathological findings; and (iv) those who provided informed consent and had complete clinical records. Exclusion criteria were: (i) individuals who had undergone cervical biopsy, conization, or chemoradiotherapy prior to elastography; (ii) those who did not subsequently receive surgical treatment; and (iii) those who did not undergo subsequent diagnosis and treatment at our institution. A total of 35 cases were enrolled (**Supplementary Figure 10**). Clinicopathological data were also collected.

#### 2.10.2 Detection of MMP1 expression in CC tissues via immunohistochemistry

Thirty-five (n=35) formalin-fixed paraffin-embedded CC tissue specimens obtained following surgical resection at the department of gynecology and obstetrics of The First Affiliated Hospital of Dalian Medical University between July and December 2023, and between January and July 2025, were retrieved. Four-micrometer tissue sections were prepared. A rabbit anti-human MMP1 monoclonal antibody (1:100, ab52631, Abcam) was used. MMP1 expression was evaluated via the IHC scoring method.

IHC scoring method: MMP1 expression was evaluated via staining intensity scores, which were defined as follows: tan (strongly positive, 3 points), brownish yellow (positive, 2 points), light yellow (weakly positive, 1 point), and negative (0 points). The percentage of positive cells was scored as follows:4 points (76%-100%), 3 points (51%-75%), 2 points (26%-50%), and 1 point (≤25%). Final IHC scores were defined as: intensity score × percentage score. Two experienced pathologists, blinded to the clinical information, independently assessed the IHC results.

#### 2.10.3 Correlation analysis of SR and MMP1 expression levels

The Mann-Whitney U test was applied to compare differences in SR between cases with MMP1 IHC scores ≤6 and >6. The association between MMP1 expression (IHC scores) and tumor stiffness (SR values) was assessed via Spearman correlation analysis.

## 3 Statistical analysis

R software (Version 4.2.1) was adopted for data analysis and visualization. The median was used for data that did not conform to a normal distribution. The Mann-Whitney U test was applied to compare two groups of data that do not conform to a normal distribution. Spearman correlation analysis was implemented to evaluate the correlation between continuous variables that do not conform to a normal distribution. Overall survival (OS) was evaluated via the KM method with log-rank testing. A threshold of P<0.05 was adopted to determine significant associations.

## 4 Results

### 4.1 Identification of MEDEGs in CESC

**Figure 1:**
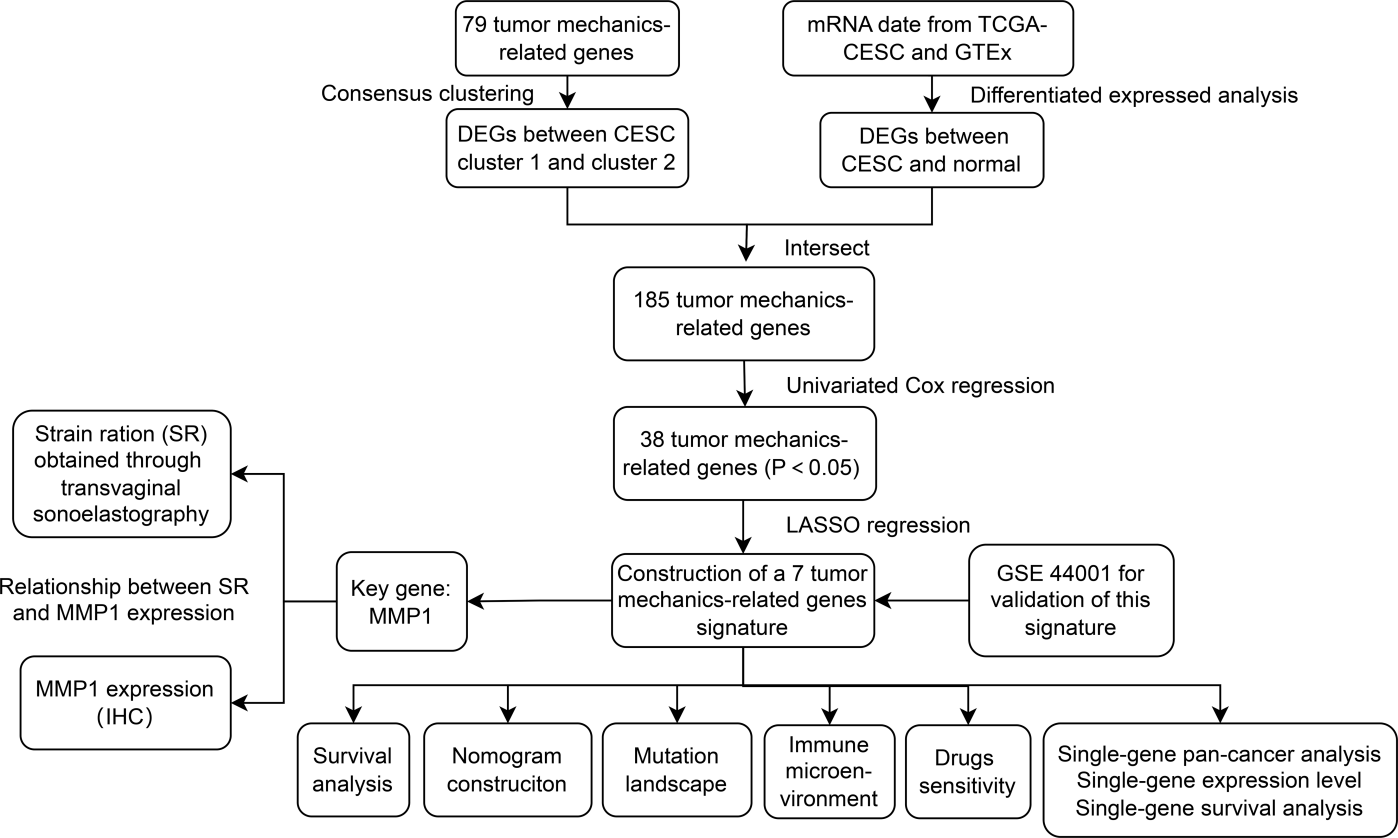
Flowchart.

**Figure 2:**
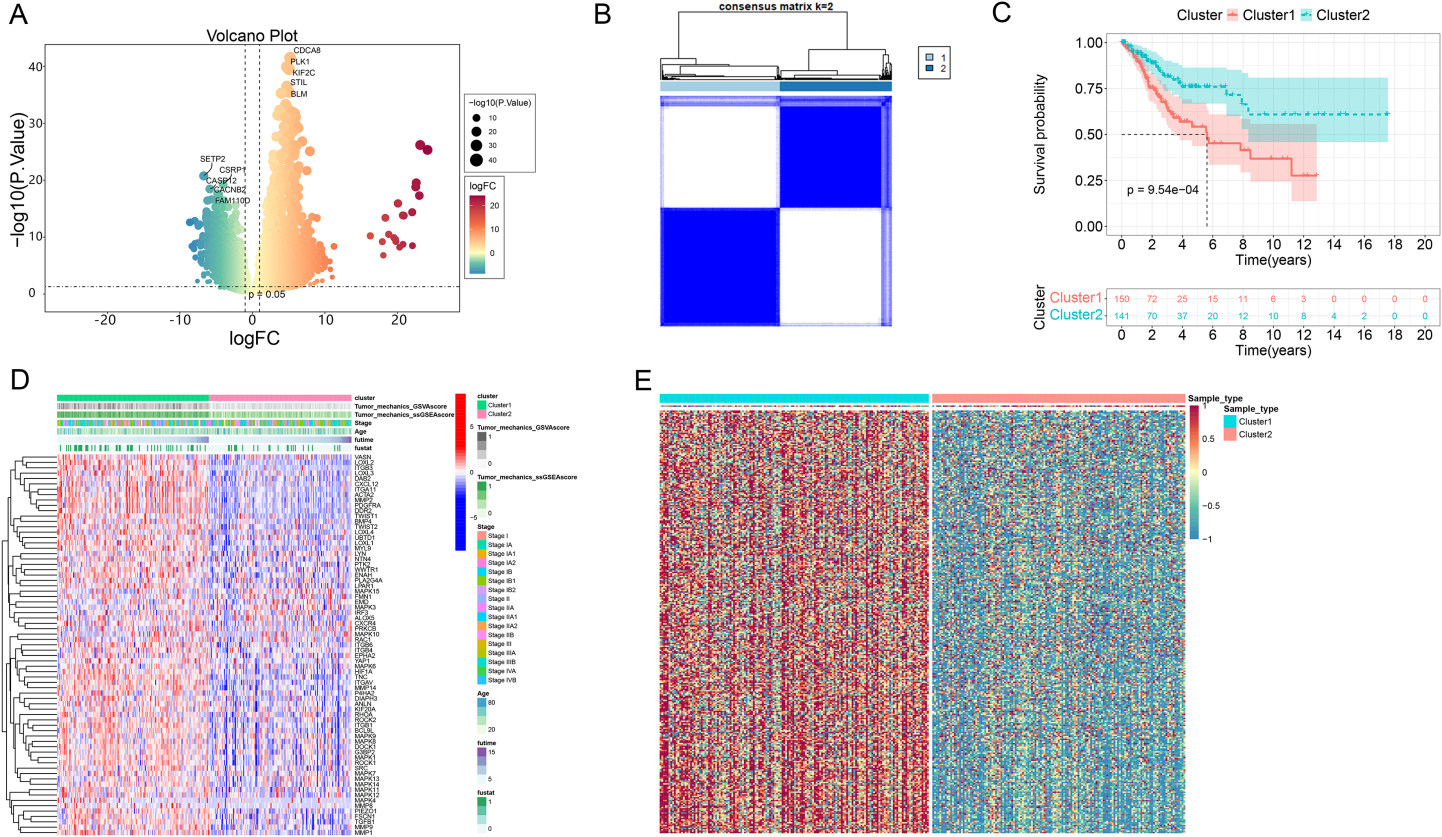
Screening of differentially expressed genes and consensus clustering of tumor mechanics-related subtypes in cervical cancer. **(A)** volcano plot of DEGs between CESC and normal cervical tissues; **(B)** consensus clustering analysis (k=2) demonstrating optimal within-group compactness and between-group separation; **(C)** survival analysis between Cluster 1 (C1) and Cluster 2 (C2); **(D)** consensus matrix for C1 and C2; **(E)** heatmap of DEGs between C1 and C2.

**Figure 1** presents the study flowchart. The initial step in identifying DEGs involved 291 CC samples (alive: 220, deceased: 71) and 91 normal samples (including 88 cervical samples from GTEx). This step revealed 9,685 DEGs (P<0.05), comprising 3,623 downregulated and 6,062 upregulated transcripts. The differentially expressed mRNA is visualized in a volcano plot (**Figure 2A**).

Consensus clustering of 291 CESC samples based on 79 TMRGs determined that two clusters, C1 and C2, exhibited optimal within-group homogeneity and between-group divergence (**Figure 2B**). KM survival analysis indicated a significant survival advantage for the C2 group compared to C1 (P<0.01, **Figure 2C**), with notable differences in expression of the TMRGs in both groups (**Figure 2D**). Comparison of the C1 and C2 groups revealed 305 MEDEGs. The mRNA expression profiles of these genes are displayed in a heat map (**Figure 2E**).

**Figure 3:**
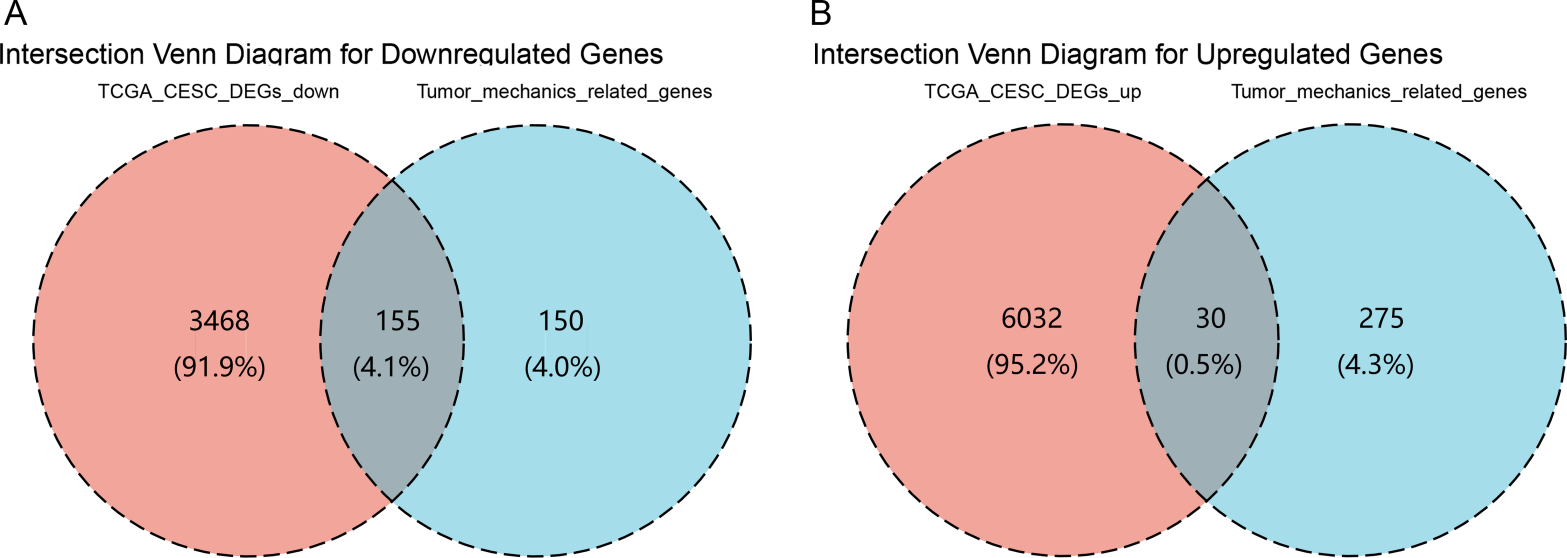
Identification of MEDEGs in CESC. **(A)** Venn diagram of MEDEGs associated with CESC (down-regulated genes); **(B)** Venn diagram of MEDEGs associated with CESC (up-regulated genes).

Intersection of DEGs between CC and normal tissues with MEDEGs yielded 185 MEDEGs in CC (**Figure 3A, B**).

### 4.2 Development and validation of a prognostic model

**Figure 4:**
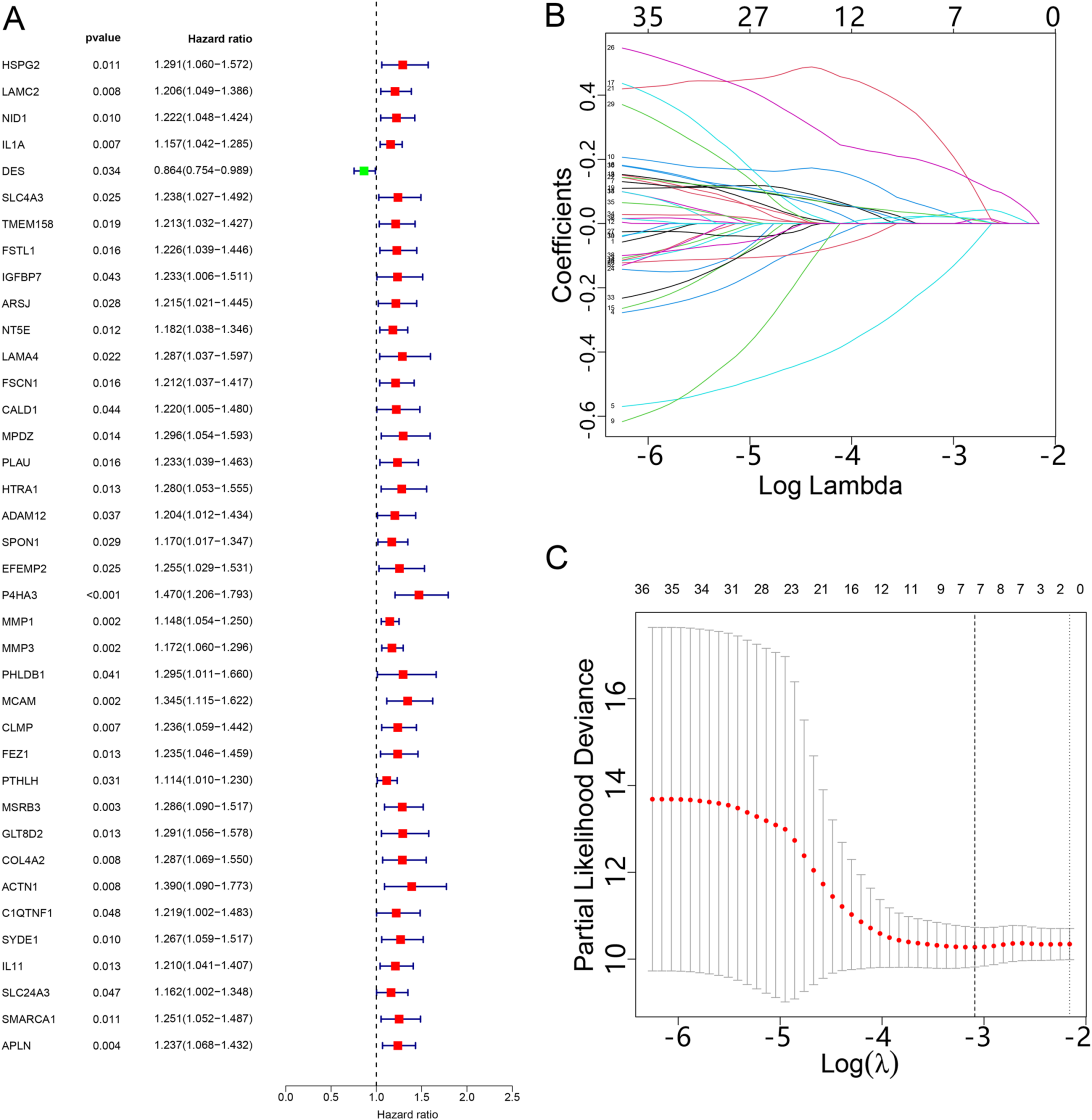
Identification of key prognosis-related tumor mechanics genes. **(A)** forest plot of prognosis-related MEDEGs in CESC; **(B)** LASSO regression of 38 candidate MEDEGs in CESC; **(C)** seven key genes selected at optimal λ (λ=7).

Univariate Cox regression analysis of 185 MEDEGs identified 38 genes correlated with prognosis (**Figure 4A**). LASSO regression (**Figure 4B**), utilizing the expression profiles of the 38 genes, identified 7 key genes (DES, ARSJ, NT5E, P4HA3, MMP1, CLMP, SMARCA1) based on the minimum λ value, comprising 1 core TMRG and 6 extended TMRGs (**Figure 4C**). Risk scores were computed as follows:

Risk score = (0.0360 × ARSJ) + (0.0287 × NT5E) + (0.2449 × P4HA3) + (0.0238 × MMP1) + (0.1738 × CLMP) + (0.0207 × SMARCA1) - (0.1576 × DES).

**Figure 5:**
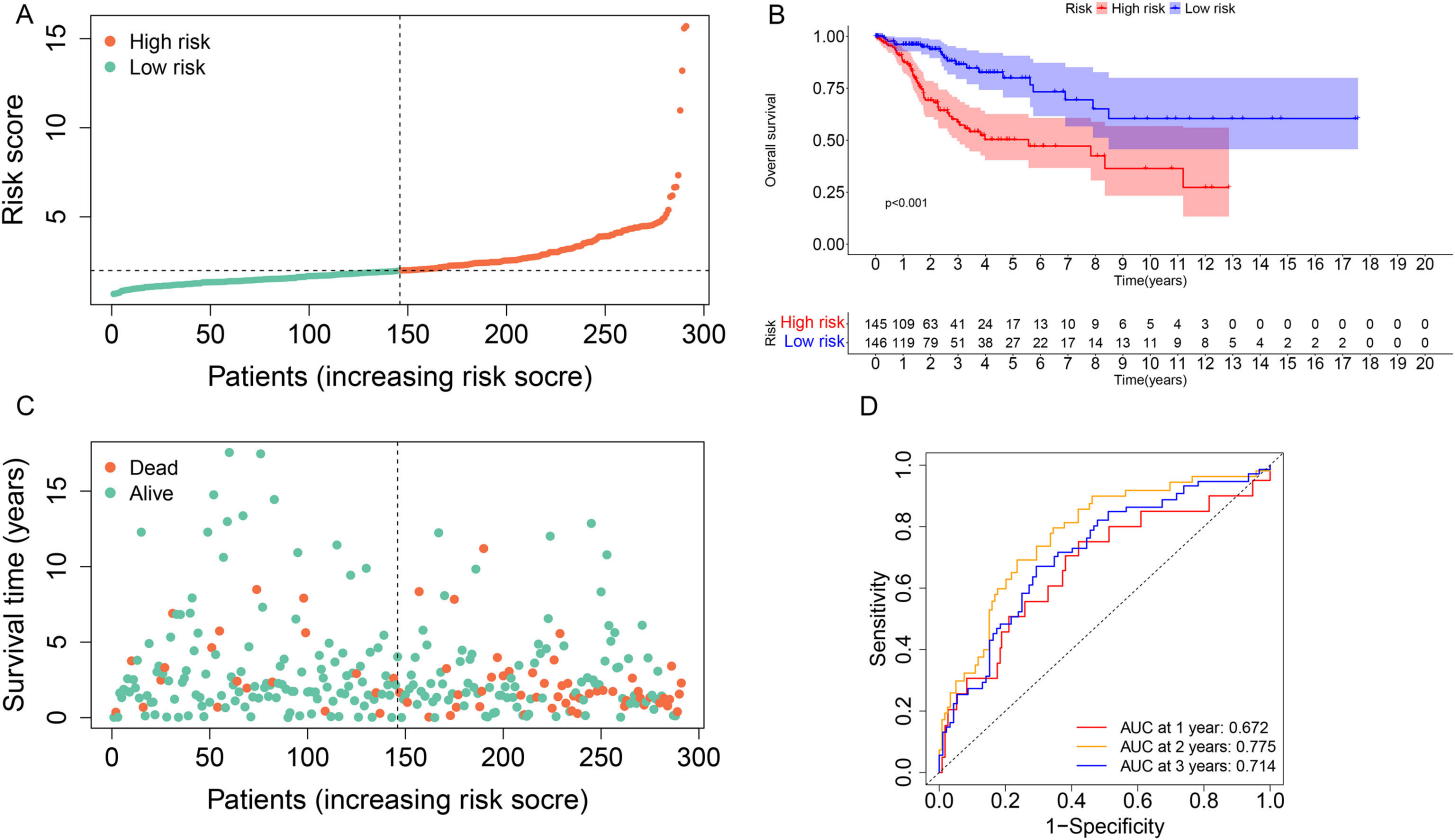
Construction of prognostic model in the TCGA cohort. **(A)** distribution of risk scores in TCGA; **(B)** KM survival analysis by risk group in TCGA; **(C)** individual survival status and risk score distribution in TCGA; **(D)** ROC curve evaluating the predictive efficacy of the 7-gene prognostic model in TCGA.

Individuals in the TCGA cohort were assigned to low- and high-risk categories according to the median risk score (**Figure 5A**). KM survival analysis revealed a markedly shorter OS in the high-risk group relative to the low-risk group (P<0.001, **Figure 5B**), and lower survival among individuals with higher risk scores (**Figure 5C**). ROC curve analysis displayed areas under the curves(AUCs) of 0.672, 0.775, and 0.714 for predicting 1-, 2-, and 3-year OS (**Figure 5D**).

Within the TCGA cohort, training and internal validation sets were established, with each set further divided into high- and low-risk groups based on the median risk score. KM analysis demonstrated that OS was significantly shorter in the high-risk group relative to the low-risk group in both the training and internal validation sets (**Supplementary Figures 1A**, **B**; both P<0.05). Time-dependent ROC analysis of the training set yielded AUCs of 0.792, 0.828, and 0.742 for predicting 1-, 2-, and 3-year OS, respectively (**Supplementary Figure 1C**). In the internal validation set, the corresponding AUCs were 0.647, 0.675, and 0.650 (**Supplementary Figure 1D**).

**Figure 6:**
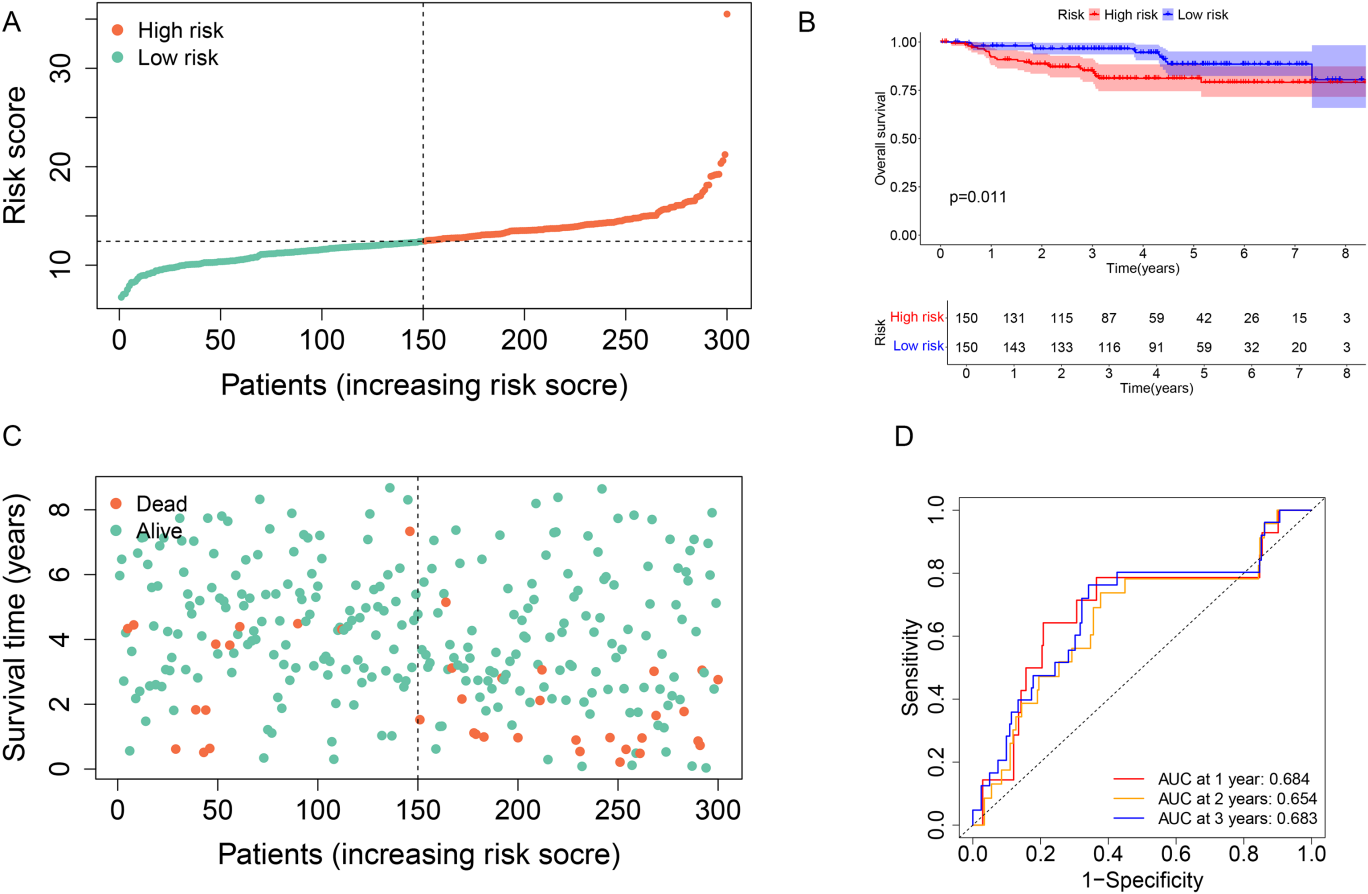
Prognostic model validation in the GEO cohort. **(A)** risk score distribution in GEO; **(B)** KM survival analysis by risk group in GEO; **(C)** individual survival status and risk score distribution in GEO; **(D)** ROC curve assessing the predictive efficacy of the 7-gene prognostic model in GEO.

Application of the same risk score formula to the GSE44001 dataset (alive: 262, deceased: 38) also stratified participants into low- and high-risk subgroups (**Figure 6A**). KM survival analysis indicated a substantially decreased OS in the high-risk group relative to the low-risk group (P = 0.011, **Figure 6B**), with lower survival among individuals with higher risk scores (**Figure 6C**). ROC curve analysis yielded AUCs of 0.684, 0.654, and 0.683 for predicting 1-, 2-, and 3-year OS (**Figure 6D**), indicating robust predictive performance.

### 4.3 Independent prognostic analysis and nomogram

**Figure 7:**
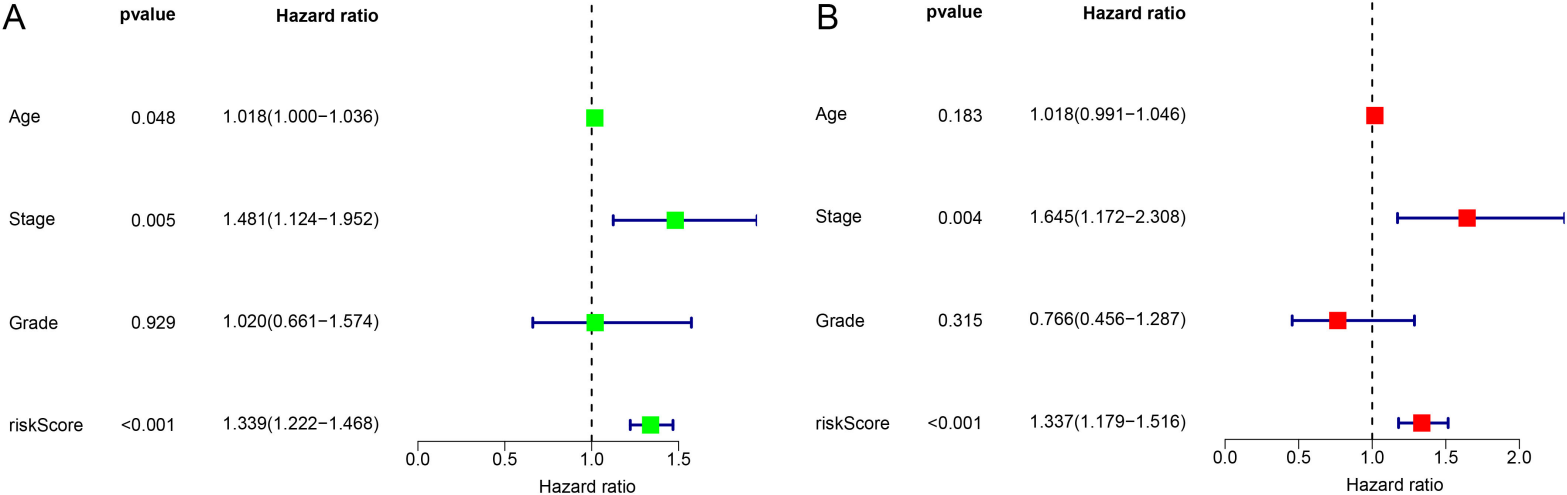
Independent prognostic analysis. **(A)** univariate Cox regression in TCGA; **(B)** multivariate Cox regression in TCGA.

Univariate Cox regression analysis in the TCGA cohort illustrated that stage (HR = 1.481, 95% CI: 1.124-1.952, P = 0.005), age (HR = 1.018, 95% CI: 1.000-1.036, P = 0.048), and risk score (HR = 1.339, 95% CI: 1.222-1.468, P<0.001) were markedly linked to OS, while histological grade was not (**Figure 7A**). Multivariate Cox regression confirmed stages and risk scores as independent prognostic factors (**Figure 7B**), indicating that the risk score independently predicts prognosis in CC individuals.

A stage-only prognostic model was built in the TCGA cohort, generating a nomogram to predict 1-, 2-, and 3-year survival probabilities (**Supplementary Figure 2A**). ROC analysis of this nomogram produced AUCs of 0.654, 0.601, and 0.575 for predicting 1-, 2-, and 3-year OS, respectively (**Supplementary Figure 2B**). The model achieved a C-index of 0.616.

**Figure 8:**
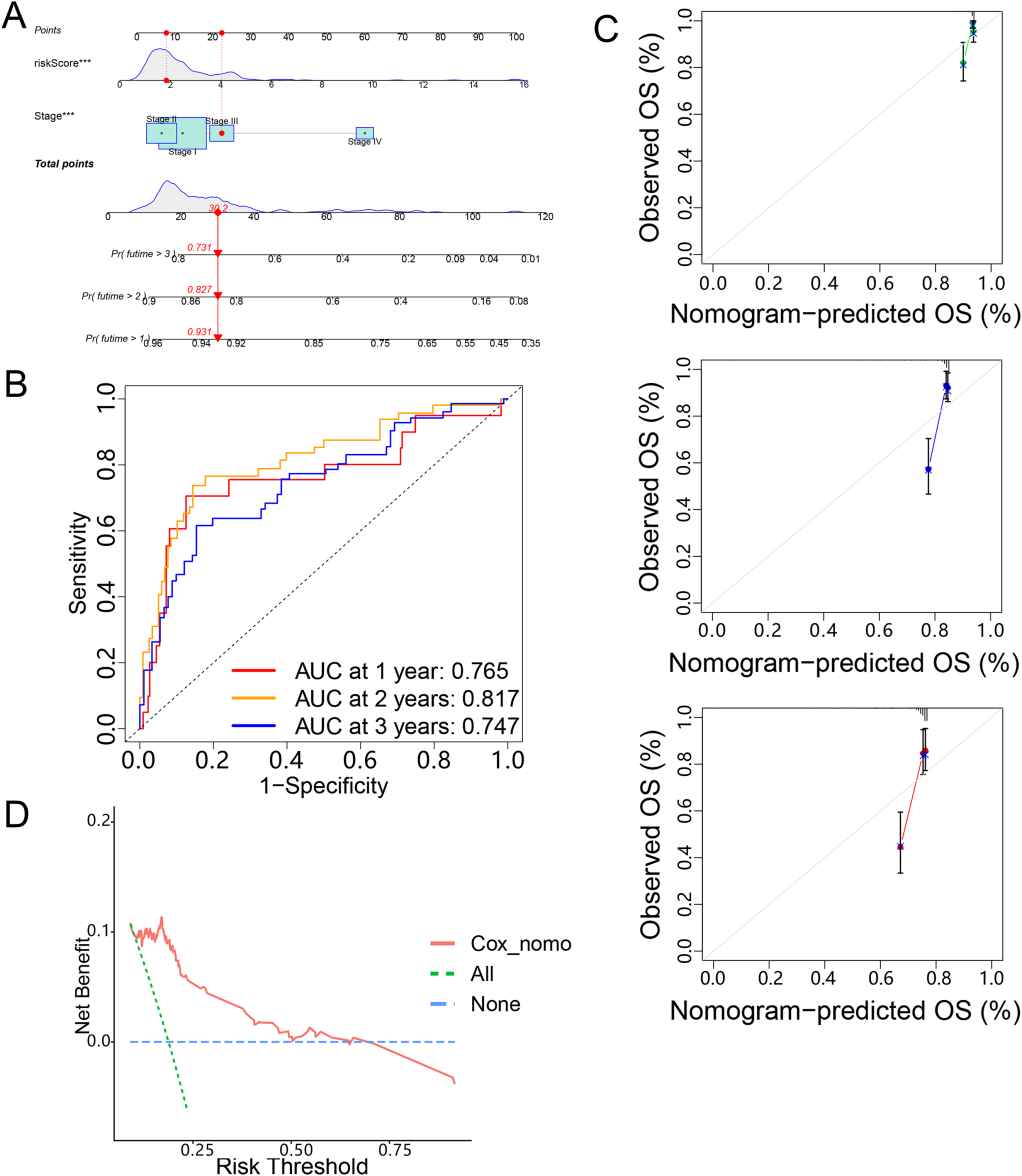
Nomogram **(A)** nomogram based on the TCGA cohort; **(B)** ROC analysis of the nomogram’s predictive accuracy; **(C)** calibration plot of the nomogram; **(D)** DCA curve.

Integrating stages and risk scores (independent prognostic factors), a predictive nomogram was created for estimating 1-, 2-, and 3-year survival probabilities in the TCGA cohort (**Figure 8A**). ROC curve analysis yielded AUCs of 0.765, 0.817, and 0.747 for predicting 1-, 2-, and 3-year OS (**Figure 8B**), with a C-index of 0.741. Calibration curves demonstrated satisfactory concordance between predicted and observed outcomes (**Figure 8C**), indicating strong predictive accuracy. Decision curve analysis (DCA) was conducted to assess the net clinical benefit of the prognostic model (**Figure 8D**).

### 4.4 Mutational landscape analysis of low- and high-risk groups

Mutational data of TCGA-CESC were analyzed, revealing differences in mutational patterns between the two risk groups. The most frequent mutations in the high-risk group involved PIK3CA (21%), TTN (21%), KMT2C (18%), MUC16 (18%), and KMT2D (14%) (**Supplementary Figure 3A**). The most frequent mutations in the other group involved TTN (46%), PIK3CA (35%), MUC4 (23%), KMT2C (22%), and FBXW7 (19%) (**Supplementary Figure 3B**). The high-risk group displayed a markedly reduced mutation rate in the oncogene PIK3CA (21%) relative to the low-risk group (35%).

### 4.5 ICI analysis

**Figure 9:**
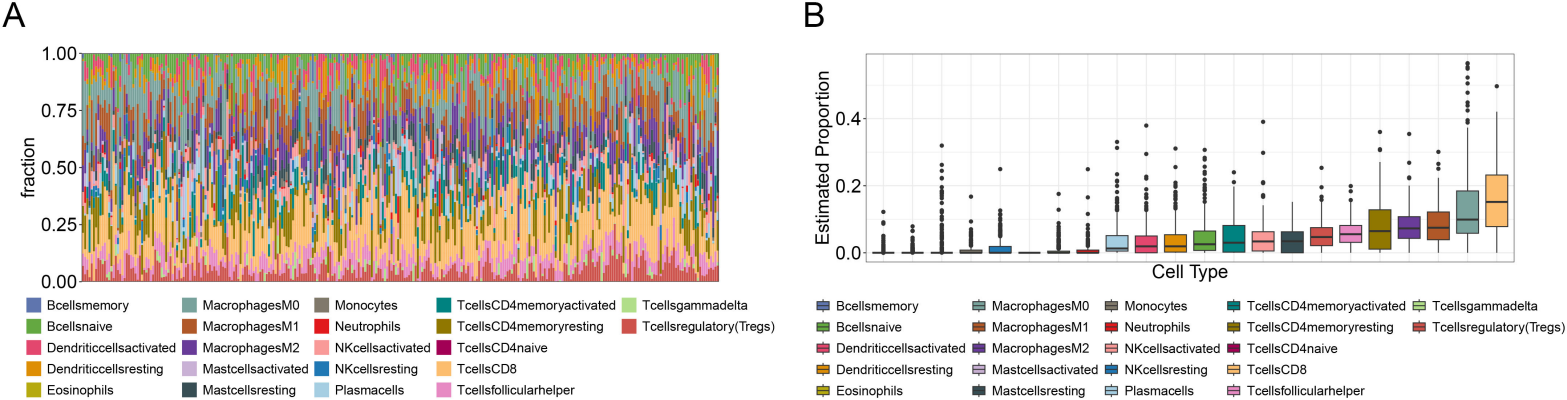
Landscape and composition of immune cell infiltration in TCGA. **(A)** ICI landscape of 22 immune cell types in TCGA; **(B)** ICI proportion of 22 immune cell types in TCGA.

**Figure 10:**
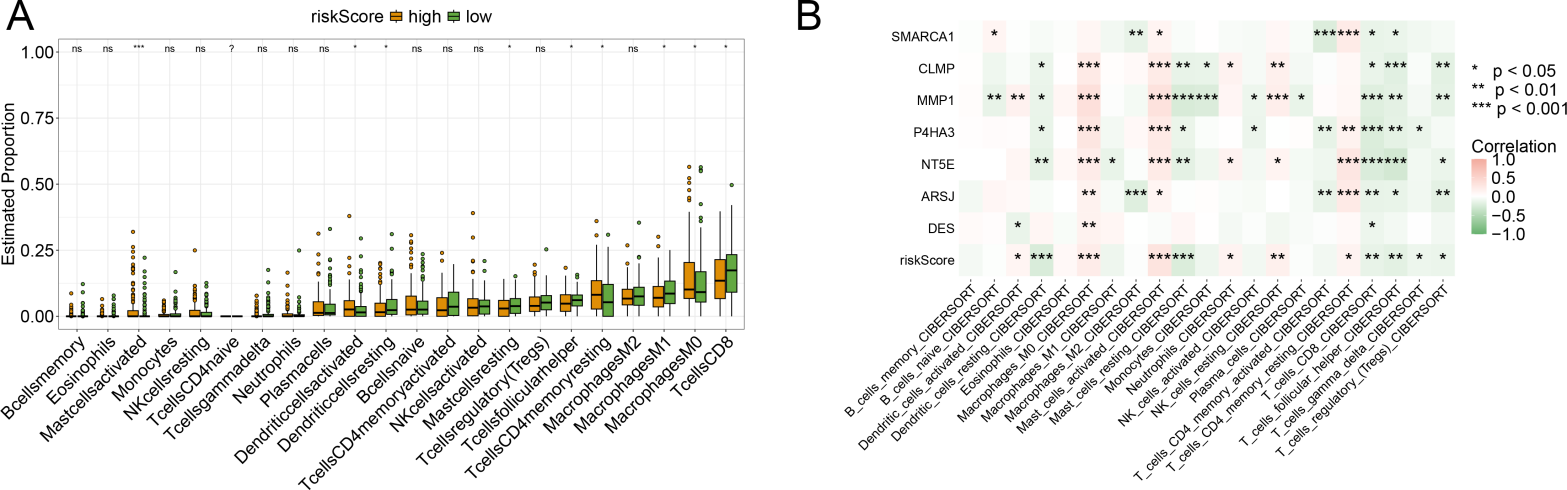
Differential immune infiltration between risk groups and its correlation with key genes. **(A)** differential infiltration levels between risk groups; **(B)** heatmap of correlations between 7 key genes/risk scores and immune cells. * represents P < 0.05, ** represents P < 0.01, and *** represents P < 0.001. "ns" means not statistically significant, that is, P ≥ 0.05. “?” indicates that statistical comparison was not applicable because this cell type showed no infiltration in either the high-risk or low-risk group.

Analysis using the CIBERSORT algorithm indicated that CESC immune infiltration was predominantly characterized by CD8+ T cells, followed by M0 macrophages (**Figure 9A, B**). Distinct levels of ICI between the two risk groups were observed. The high-risk group demonstrated increased abundance of activated dendritic cells, activated mast cells, resting memory CD4+ T cells, and M0 macrophages, while the low-risk group exhibited higher levels of resting dendritic cells, resting mast cells, follicular helper T cells, M1 macrophages, and CD8+ T cells (**Figure 10A**). A heatmap displaying the correlation between the seven key genes, risk score, and ICI patterns (**Figure 10B**) indicated that immune infiltration differences might contribute to the distinct prognoses of the two risk groups.

### 4.6 Clinical correlation analysis

Correlation analysis of the prognostic model with clinical characteristics illustrated that risk scores were significantly elevated among individuals aged ≤60 years relative to those aged >60 years (P<0.05, **Supplementary Figure 4A**). Risk scores were also significantly elevated among individuals with grade G3 compared to grade G2 (P<0.05, **Supplementary Figure 4B**). No significant relationship between risk score and disease stage (P>0.05, **Supplementary Figure 4C**) was observed.

### 4.7 Drug sensitivity analysis

To assess the potential response to drug therapy in CESC, the difference in drug sensitivity between the low- and high-risk groups was analyzed. The high-risk group showed greater sensitivity to docetaxel, cytarabine, and elesclomol. The low-risk group exhibited greater sensitivity to MP470, GSK690693, and Navitoclax. Additionally, drug sensitivity was found to be correlated with risk scores (|correlation coefficient|>0.3, P<0.001, **Supplementary Figure 5**). This provides potential leads for individualized pharmacotherapy in CC.

### 4.8 Single-gene pan-cancer analysis

Differential expression analysis of the seven core genes across 33 cancer types showed elevated MMP1 expression in 18 cancer types and lower expression in 2 cancer types (P<0.05, **Supplementary Figure 6A**). DES was downregulated in 18 cancer types and upregulated in 1 cancer type (P<0.05). Expression differences of the remaining genes were cancer-specific (**Supplementary Figures 7A–F**).

Differential expression analysis of seven core genes in 15 cancer types relative to matched normal tissues indicated elevated expression of MMP1 and P4HA3 (MMP1 expression shown in **Supplementary Figure 6B**) and decreased expression of DES and CLMP. Expression differences of ARSJ, NT5E, and SMARCA1 were cancer-specific (**Supplementary Figures 8A–F**).

GO enrichment analysis of MMP1 and its co-expressed genes demonstrated a positive correlation with cell response to lipopolysaccharide, biological stimulation response, and regulation of extrinsic apoptotic signaling pathways. A negative correlation was observed with fatty acid catabolism, monocarboxylic acid catabolism, and regulation of the vitamin D receptor signaling pathway (**Supplementary Figure 6C**).

### 4.9 Single-gene expression and survival analysis

In TCGA-CESC, expression of DES, NT5E, CLMP, and SMARCA1 was substantially lower in tumor tissue relative to normal tissue (P<0.05), while MMP1 expression was markedly elevated in tumor tissue (P<0.05, **Supplementary Figure 6D**).

Single-gene survival analysis based on TCGA-CESC data (categorized by the optimal threshold) illustrated that lower expression of MMP1, ARSJ, NT5E, P4HA3, CLMP, and SMARCA1 correlated with significantly improved OS (P<0.05, MMP1 survival curve presented in **Supplementary Figure 6E**), while lower DES expression correlated with significantly decreased OS (P<0.05, **Supplementary Figures 9A–F**).

### 4.10 Clinical sample validation

The SE of 35 CC individuals revealed that the SR, reflecting tissue hardness, ranged from 2.03 to 6.22 across all lesions. IHC demonstrated that MMP1 was predominantly expressed in the cytoplasm of CC cells. One case was negative, and the remaining 34 exhibited varying degrees of positive expression. The flowchart of participant enrollment is presented in **Supplementary Figure 10**. For each case, clinicopathological data were compiled, including age, pathological types, histological grade, focus size, depth of invasion, vascular invasion, nerve invasion, and node metastasis. Peripheral blood squamous cell carcinoma antigen (SCC-Ag) levels and the SR determined by SE were also documented. **Table 1** shows the specific information of each case.

**Table 1:** Clinical and pathological characteristics of patients with cervical cancer.

Cases	Age	Pathological types of cervical cancer	Histological grade	Focus size	Depth of invasion	Is there any vascular invasion?	Is there nerve invasion?	Is there lymph node metastasis?	Other metastatic foci in pathological specimens	Squamous cell carcinoma antigen (SCC-Ag) (0-1.8)	Stain ratio	MMP1 IHC score
1	50	Squamous carcinoma	Moderate differentiation	2.8×2cm	>1/2 of the full thickness	Yes	Yes	No	No	1.8	4.27	8
2	32	Adenocarcinoma	Low differentiation	3×2.5×1.5cm	≤1/2 of the full thickness	No	No	No	No	0.66	3.35	4
3	43	Adenocarcinoma	Low differentiation	1.2×1cm	≤1/2 of the full thickness	No	No	No	No	0.42	3.09	12
4	37	Squamous carcinoma	Low differentiation	3×3cm	>1/2 of the full thickness	Yes	Yes	Yes	Involving the vaginal fornix	1.41	5.41	4
5	37	Adenocarcinoma	Low differentiation	7×6cm	>1/2 of the full thickness	No	No	No	Metastasis observed in the right ovary	0.75	4.08	12
6	44	Squamous carcinoma	Moderate differentiation	3×1.8×1.7cm	>1/2 of the full thickness	Yes	No	No	No	0.82	2.99	12
7	43	Squamous carcinoma	Low differentiation	4×3.8×1.2cm	>1/2 of the full thickness	Yes	Yes	Yes	No	4.39	3.22	4
8	37	Squamous carcinoma	Low differentiation	2×1.8×0.6cm	≤1/2 of the full thickness	Yes	No	No	No	1.08	2.95	4
9	51	Squamous carcinoma	Moderately low differentiation	4.2×1.5×0.8	About 1/2 of the full thickness	Yes	No	No	No	2.1	3.09	2
10	51	Squamous carcinoma	Low differentiation	2.8×2.5×0.6cm	≤1/2 of the full thickness	No	No	No	No	4.23	2.03	3
11	53	Focal adenocarcinoma with squamous carcinoma	NA	2.5×2×0.5cm	≤1/2 of the full thickness	Yes	No	No	No	0.69	3.16	3
12	51	Squamous carcinoma	Moderately low differentiation	2×1×0.7cm	>1/2 of the full thickness	Yes	No	Yes	A tumor thrombus found in the right parametrial vein	0.74	3.36	4
13	52	Squamous carcinoma	Moderately low differentiation	3.8×3.2×2.2cm	About of the full thickness	No	No	No	No	4.69	4.52	12
14	46	Squamous carcinoma	Moderate differentiation	1.5×1×0.5cm	≤1/2 of the full thickness	No	No	No	Endometrium with polyps and active focal hyperplasia	1.05	3.97	8
15	59	Adenocarcinoma	low differentiation	3×2cm	>1/2 of the full thickness	Yes	No	Yes	Metastatic foci found in the colon	0.67	2.94	4
16	55	Squamous carcinoma	Moderately low differentiation	2.4×2×2cm	>1/2 of the full thickness	Yes	No	No	No	1.43	3.69	0
17	69	Adenocarcinoma	low differentiation	6.6×6×2.4cm	About of the full thickness	Yes	Yes	No	Involving the endometrium and the vaginal fornix	0.89	4.28	8
18	51	Squamous carcinoma	Low differentiation	2×1.5cm	>1/2 of the full thickness	Yes	No	Yes	No	1.56	3.46	2
19	43	Squamous carcinoma	Low differentiation	2.2×1.8×1.2cm	>1/2 of the full thickness	Yes	No	No	No	0.72	3.14	4
20	55	Squamous carcinoma with a small amount of adenocarcinoma(<10%)	Low differentiation	2.2×1.5cm	>1/2 of the full thickness	Yes	Yes	No	No	2.31	3.93	4
21	42	Squamous carcinoma	Low differentiation	4.5×3.5cm	About of the full thickness	Yes	Yes	Yes	Involving the vaginal fornix, parametrium and uterine body	1.46	4.52	12
22	37	Squamous carcinoma	Low differentiation	5×4×2.5cm	>1/2 of the full thickness	Yes	Yes	Yes	No	7.7	5.5	8
23	58	Squamous carcinoma	Moderately low differentiation	4×2cm	>1/2 of the full thickness	Yes	Yes	Yes	No	7.37	6.22	8
24	45	Adenocarcinoma	low differentiation	4×2.5×1cm	≤1/2 of the full thickness	No	No	No	No	0.76	5.72	8
25	59	Squamous carcinoma	Moderately low differentiation	5×2.5×2cm	About of the full thickness	Yes	Yes	Yes	Involving the vaginal fornix	2.54	5.38	4
26	64	Squamous carcinoma	Moderately differentiation	2×1.5×1cm	>1/2 of the full thickness	Yes	No	No	Involving the vaginal fornix	0.55	3.83	4
27	55	Squamous carcinoma	Moderately low differentiation	4.5×2.5×2.3cm	About of the full thickness	Yes	Yes	Yes	Involving the vaginal fornix	40.96	4.13	8
28	42	Squamous carcinoma	low differentiation	2.2×2×0.5cm	<1/2 of the full thickness	No	No	No	No	0.41	4.16	4
29	66	Squamous carcinoma	Moderately low differentiation	3×2.2×1.5cm	>1/2 of the full thickness	Yes	No	Yes	No	2.13	5.22	8
30	67	Squamous carcinoma	low differentiation	5×2cm	>1/2 of the full thickness	Yes	Yes	Yes	Involving the vaginal fornix, parametrium and metastasis observed in the left ovary	16.11	4.5	8
31	73	Squamous carcinoma	low differentiation	4×3×1cm	the full thickness	Yes	Yes	No	Involving the vaginal body	1.72	4.5	4
32	55	Squamous carcinoma	low differentiation	5.5×2.8×0.5cm	the full thickness	Yes	Yes	Yes	No	1.25	5.82	4
33	66	Squamous carcinoma	low differentiation	6×4×3cm	the full thickness	Yes	No	No	No	25.4	3.82	4
34	62	Squamous carcinoma	Moderately low differentiation	5.5×5.3×3.5cm	the full thickness	Yes	No	Yes	No	1.46	6.02	8
35	51	Squamous carcinoma	low differentiation	1.6×0.5×0.8cm	<1/2 of the full thickness	No	No	No	No	0.65	3.32	3

Spearman correlation analysis revealed a moderate positive link between MMP1 IHC scores and SR (r=0.418, P = 0.012). The high IHC score group (>6, n=15) demonstrated a higher mean SR than the low-score group (≤6, n=20) (4.5 vs. 3.41, P = 0.008).

**Figure 11:**
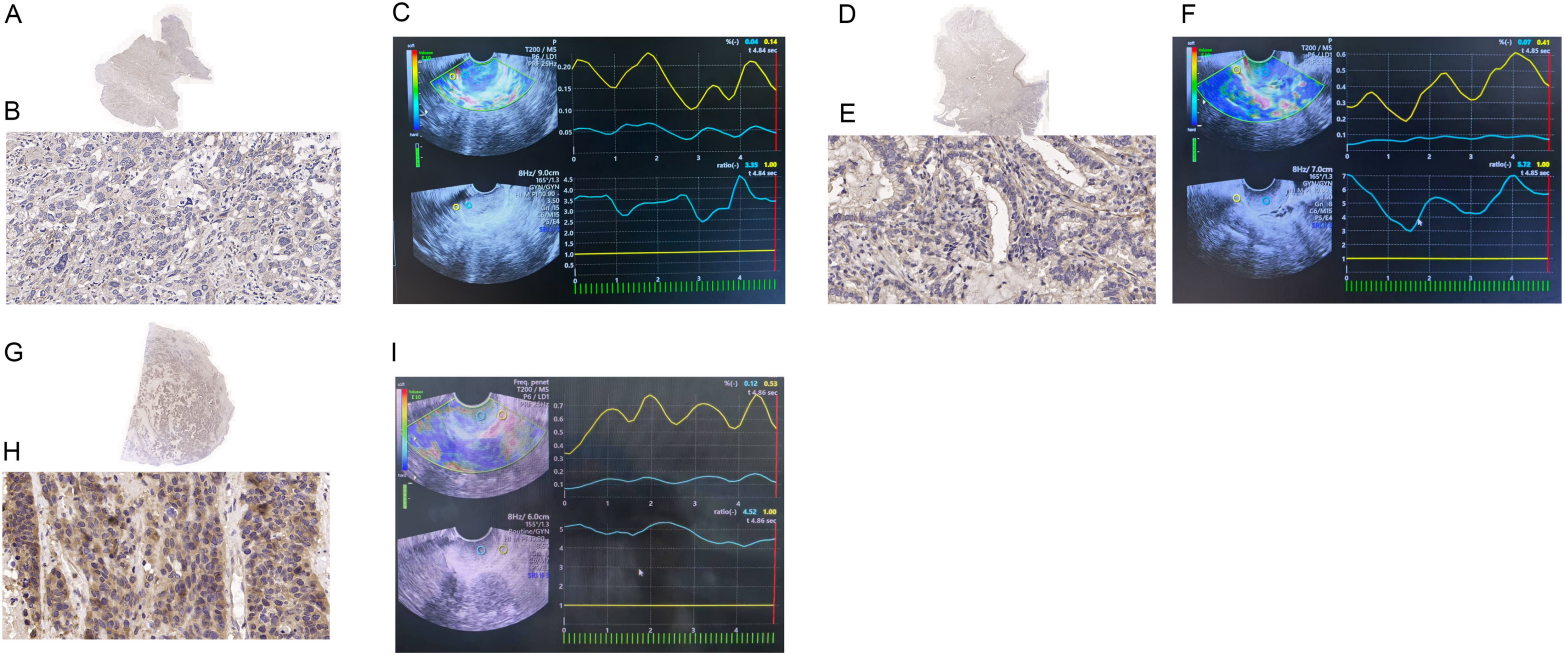
SR and MMP1 expression in clinical CC samples. **(A)** IHC staining of MMP1 in Case 1; **(B)** higher-magnification view (×40) of **(A)** (IHC score: 4); **(C)** SE image of Case 1 (SR = 3.35, indicating low stiffness); **(D)** IHC staining of MMP1 in Case 2; **(E)** higher-magnification view (×40) of **(D)** (IHC score: 8); **(F)** SE image of Case 2 (SR = 5.72, indicating high stiffness); **(G)** IHC staining of MMP1 in Case 3; **(H)** higher-magnification view (×40) of **(G)** (IHC score: 12); **(I)** SE image of Case 3 (SR = 4.52, indicating high stiffness).

Typical cases displayed that the patient in **Figure 11A–C** had adenocarcinoma with low MMP1 expression (score of 4 points) and a corresponding SR of 3.35 with low stiffness. The patient in **Figure 11D–F** had adenocarcinoma with a higher expression of MMP1 (score of 8 points) and a corresponding SR of 5.72 with high stiffness. The patient in **Figure 11G–I** had squamous carcinoma with a high expression of MMP1 (score of 12 points) and a corresponding SR of 4.52 with high stiffness.

## 5 Discussion

Tumor mechanical properties, particularly tumor stiffness, which acts as an indicator of the mechanical status of the TME, are tightly linked to tumor progression, development, and treatment response. Biomechanical properties of the ECM are altered even prior to tumor formation, as the increase in ECM stiffness and the changes in its structure are important factors in the pathogenesis of diverse malignancies (14, 15). Aberrant ECM mechanical properties can activate signaling pathways that promote the proliferation of tumor cells. For instance, increased ECM stiffness can promote cell proliferation by activating the ERK signaling pathway (16) and can enhance the tumor invasion and metastasis by inducing epithelial-mesenchymal transition (EMT) (17). Elevated tumor stiffness is the direct mechanical phenotype of these pathological processes. Although existing studies have suggested the prognostic value of tumor mechanical gene sets (13), the expression of TMRGs in CC and the correlation between their expression and tumor stiffness have not been specifically examined.

The present study revealed that more than half of the TMRGs (185/305, 60.7%) were dysregulated in CESC, indicating a potential functional contribution of these genes to CESC progression. Using the TCGA cohort, this study constructed a model, plotted ROC curves, and obtained 1-, 2-, and 3-year AUCs of 0.672, 0.775, and 0.714. The respective AUCs of the validation cohort GSE44001 were 0.684, 0.654, and 0.683. The AUCs of the nomogram were 0.765, 0.817, and 0.747. The C-index of the model was 0.741. These results highlight the reliable predictive ability of the model for CESC individuals. Subsequent immune infiltration and drug sensitivity analyses illustrated significant differences between the low- and high-risk groups.

The TME exerts a profound influence on tumor progression and therapeutic responsiveness. The present study conducted an immune infiltration analysis comparing low- and high-risk groups and revealed differences in ICI. The high-risk group exhibited elevated levels of M0 macrophages, resting memory CD4+ T cells, activated mast cells, and activated dendritic cells. The low-risk group exhibited higher levels of resting dendritic cells, follicular helper T cells, resting mast cells, M1 macrophages, and CD8+ T cells. Furthermore, the risk score was significantly linked to the tumor immune microenvironment. Activated mast cells and M0 macrophages were positively linked to risk scores (P<0.001). M0 macrophages possess tumor-promoting and immunosuppressive properties. Activated mast cells can promote angiogenesis and tissue remodeling, thereby facilitating tumor metastasis and suppressing anti-tumor immunity. These findings highlight clear variations in the tumor immune microenvironment between low- and high-risk groups.

Cancer-associated fibroblasts (CAFs), a heterogeneous cell population, are widely present in various solid tumors (18). CAFs promote the growth, proliferation, and metastasis of tumors not only by interacting with immune cells to exert immunosuppressive functions but also by participating in stromal deposition and remodeling. CAFs modulate the mechanical properties of the TME by synthesizing and depositing collagen (19). This suggests that tumor-intrinsic mechanical alterations could be intimately linked to matrix remodeling and aberrant immune infiltration. The present investigation revealed pronounced differences in the immune infiltration and escapes between the high- and low-expression groups of tumor mechanical genes in CC.

MMP1 belongs to the matrix metalloproteinase family of interstitial collagenases. It is an important component involved in ECM degradation and protease hydrolysis.

Enrichment analysis of MMP1 and its co-expressed genes revealed their involvement in inflammation-related pathways, including the cellular response to lipopolysaccharide and the cellular response to molecules of bacterial origin. Within the TME, inflammatory mediators such as HMGB1 are closely implicated in the initiation and progression of tumors. HMGB1-mediated autophagy may potentiate chemoresistance in diverse malignancies (20). Inflammatory factors can activate normal fibroblasts to transform them into CAFs (21), driving tumor stromal remodeling to a certain degree. This observation further suggests a potential coupling mechanism between inflammatory responses and mechanically relevant alterations arising from matrix remodeling.

Prior investigations have linked MMP1 to the progression of several malignancies—most notably bladder (22), prostate (23), and gastric cancers (24)—where its expression correlates closely with prognosis. Silencing MMP1 attenuates the proliferative, migratory, and invasive capacity of CC cell lines, and MMP1 appears to drive the growth and metastasis of CC tissues via EMT in these cell lines (17). The present findings suggest that elevated MMP1 expression may be associated with an unfavorable prognosis in CC. Clinical validation revealed a positive correlation between lesionalMMP1expression levels and SR (P<0.05); the SR in the high MMP1 expression group exceeded that in the low expression group (P<0.05). As a preliminary observation, MMP1 showed differential expression across CC tissues with varying stiffness properties. MMP1 may thus represent a potential accompanying marker of ECM remodeling and inflammatory processes. This offers a tentative clue for further exploration of the relationship between MMP1 and tumor-intrinsic mechanical alterations.

Several limitations merit consideration. First, this investigation constitutes an exploratory bioinformatics analysis. Although the prognostic model was derived from large-scale public databases, its clinical robustness requires further validation in larger, multicenter prospective cohorts. Stratification by distinct pathological subtypes of CC was not undertaken. Future large-scale prospective studies should address this limitation. Second, the clinical validation portion is constrained by a relatively modest sample size from a single center. The SE-based assessment of tumor stiffness also involves a degree of subjectivity. Third, while a preliminary link was observed between MMP1 and tumor stiffness, this association still requires more rigorous support through continuous-variable correlation analyses and functional experiments to elucidate the potential molecular mechanisms. Consequently, the current results should be interpreted as a preliminary framework for future biomechanical investigations in CC.

In summary, this study focused on tumor mechanical properties and developed a CC prognostic model based on TMRGs. The findings suggest a preliminary link between MMP1 expression and tumor stiffness, which may support the future development of new prognostic biomarkers and tumor stiffness-informed clinical evaluation.

## Data Availability

Publicly available datasets were analyzed in this study. This data can be found here: The transcriptomic data were retrieved from public repositories (UCSC Xena and GEO). The code is freely accessible via the following link: https://github.com/drdyzhanglu/Gynecology. The clinical data will be made available from the corresponding author, Yuhong Shang, upon reasonable request.

## Supplementary material

Supplementary Figure 1 Kaplan-Meier and ROC curve analysis of the prognostic model in the TCGA internal training and validation sets. **(A)** Kaplan-Meier survival curves of high- and low-risk groups in the TCGA internal training set. **(B)** Kaplan-Meier survival curves of high- and low-risk groups in the TCGA internal validation set. **(C)** Time-dependent ROC curves for predicting 1-, 2-, and 3-year OS in the TCGA internal training set. **(D)** Time-dependent ROC curves for predicting 1-, 2-, and 3-year OS in the TCGA internal validation set.

Supplementary Figure 2 Nomogram and ROC curves of the stage-only model based on the TCGA cohort. **(A)** Nomogram based solely on clinical stage for predicting 1-, 2-, and 3-year OS. **(B)** Time-dependent ROC curves of the stage-only model for 1-, 2-, and 3-year OS prediction.

Supplementary Figure 3 Mutational landscape by risk group. **(A)** Mutation profile of high-risk individuals; **(B)** Mutation profile of low-risk individuals.

Supplementary Figure 4 Clinical correlation analysis. **(A)** Association of risk scores with age; **(B)** Association of risk scores with histologic grade; (C) Association of risk scores with stage.

Supplementary Figure 5 Scatter plots depicting the correlation between risk score and drug sensitivity (top 3 genes in positive correlation and negative correlation).

Supplementary Figure 6 Pan-cancer analysis of MMP1, single-gene expression levels, and survival analysis. **(A)** MMP1 expression in tumor vs. normal tissues (pan-cancer); **(B)** MMP1 expression in tumor vs. adjacent tissues (pan-cancer); (C) GO enrichment of MMP1 co-expressed genes; **(D)** Expression levels of core genes in CESC; **(E)** Single-gene survival analysis of MMP1.

Supplementary Figure 7 Pan-cancer differential expression analysis of core genes between tumor and normal tissues. **(A)** Expression of DES in pan-cancer tumor vs. normal tissues; **(B)** Expression of ARSJ in pan-cancer tumor vs. normal tissues; **(C)** Expression of CLMP in pan-cancer tumor vs. normal tissues; **(D)** Expression of NT5E in pan-cancer tumor vs. normal tissues; **(E)** Expression of P4HA3 in pan-cancer tumor vs. normal tissues; **(F)** Expression of SMARCA1 in pan-cancer tumor vs. normal tissues.

Supplementary Figure 8 Pan-cancer differential expression analysis of core genes between tumor and adjacent tissues. **(A)** Expression of DES in pan-cancer tumor vs. tumor-adjacent tissues; **(B)** Expression of ARSJ in pan-cancer tumor vs. tumor-adjacent tissues; **(C)** Expression of CLMP in pan-cancer tumor vs. tumor-adjacent tissues; **(D)** Expression of NT5E in pan-cancer tumor vs. tumor-adjacent tissues; **(E)** Expression of P4HA3 in pan-cancer tumor vs. tumor-adjacent tissues; **(F)** Expression of SMARCA1 in pan-cancer tumor vs. tumor-adjacent tissues.

Supplementary Figure 9 Single-gene survival analysis of core genes; **(A)** DES; **(B)** ARSJ; **(C)** CLMP; **(D)** NT5E; **(E)** P4HA3; **(F)** SMARCA1.

Supplementary Figure 10 Flowchart of the clinical case screening process.
