# Supplementary figures and images for: Preliminary construction and validation of a prognostic prediction model for cervical cancer based on tumor mechanics-related genes
Source: Front Oncol. 2026 Jun 3;16:1841456. doi: 10.3389/fonc.2026.1841456 (PMC13272372; doi:10.3389/fonc.2026.1841456)

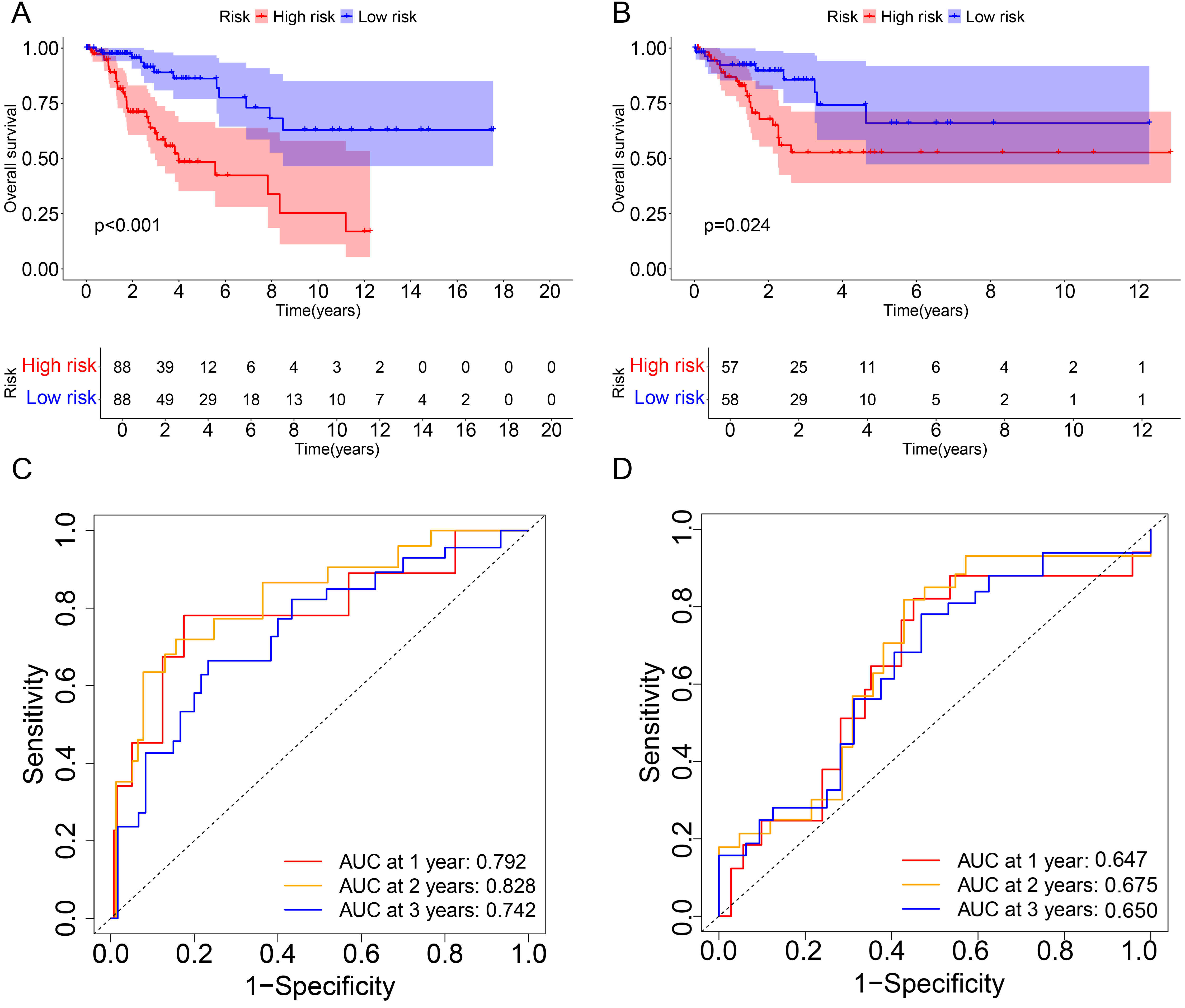

Supplement: Supplementary Figure 1 — Kaplan-Meier and ROC curve analysis of the prognostic model in the TCGA internal training and validation sets. (A) Kaplan-Meier survival curves of high- and low-risk groups in the TCGA internal training set. (B) Kaplan-Meier survival curves of high- and low-risk groups in the TCGA internal validation set. (C) Time-dependent ROC curves for predicting 1-, 2-, and 3-year OS in the TCGA internal training set. (D) Time-dependent ROC curves for predicting 1-, 2-, and 3-year OS in the TCGA internal validation set. [file Image1.tif]

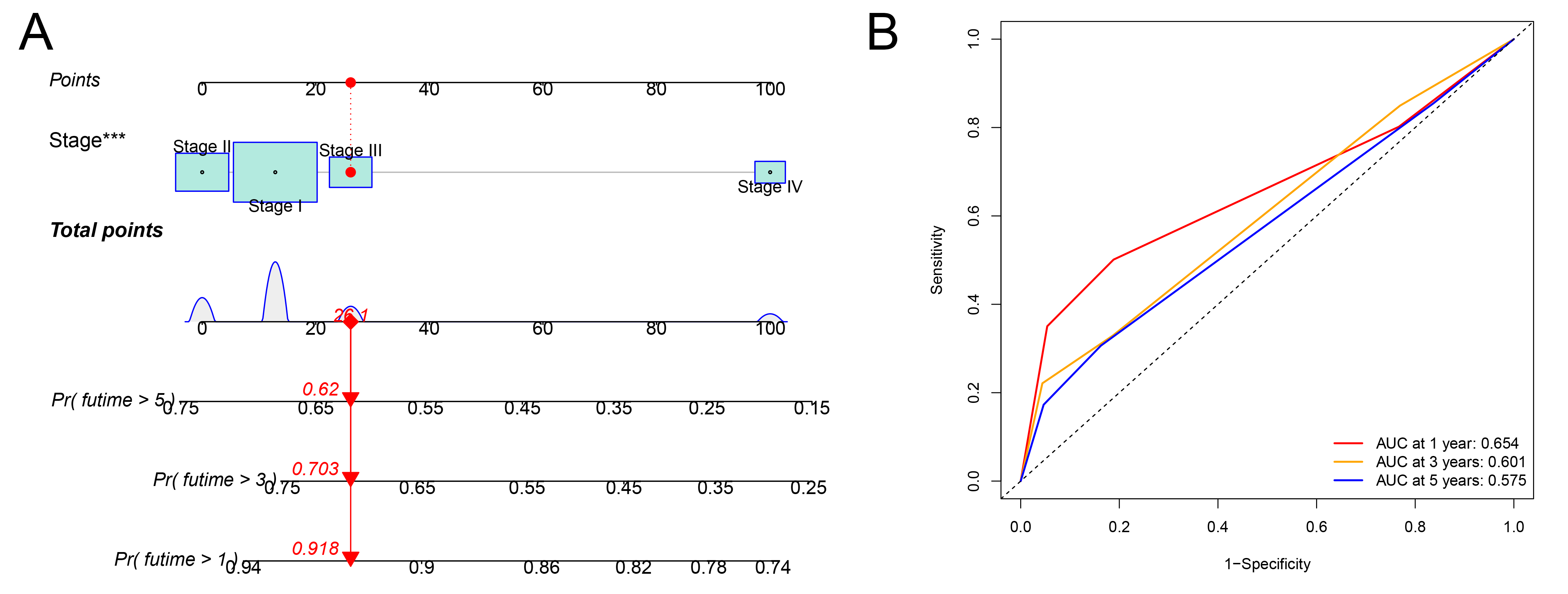

Supplement: Supplementary Figure 2 — Nomogram and ROC curves of the stage-only model based on the TCGA cohort. (A) Nomogram based solely on clinical stage for predicting 1-, 2-, and 3-year OS. (B) Time-dependent ROC curves of the stage-only model for 1-, 2-, and 3-year OS prediction. [file Image2.tif]

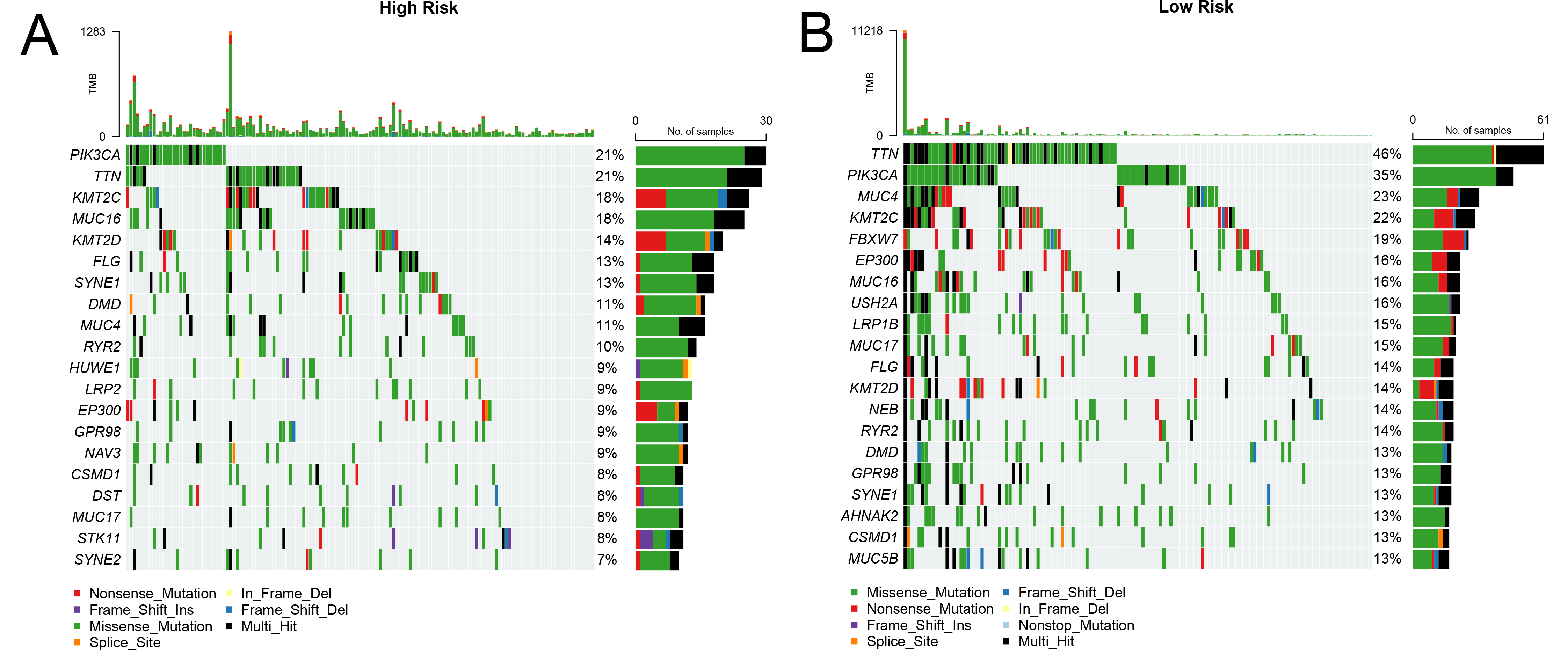

Supplement: Supplementary Figure 3 — Mutational landscape by risk group. (A) Mutation profile of high-risk individuals; (B) Mutation profile of low-risk individuals. [file Image3.tif]

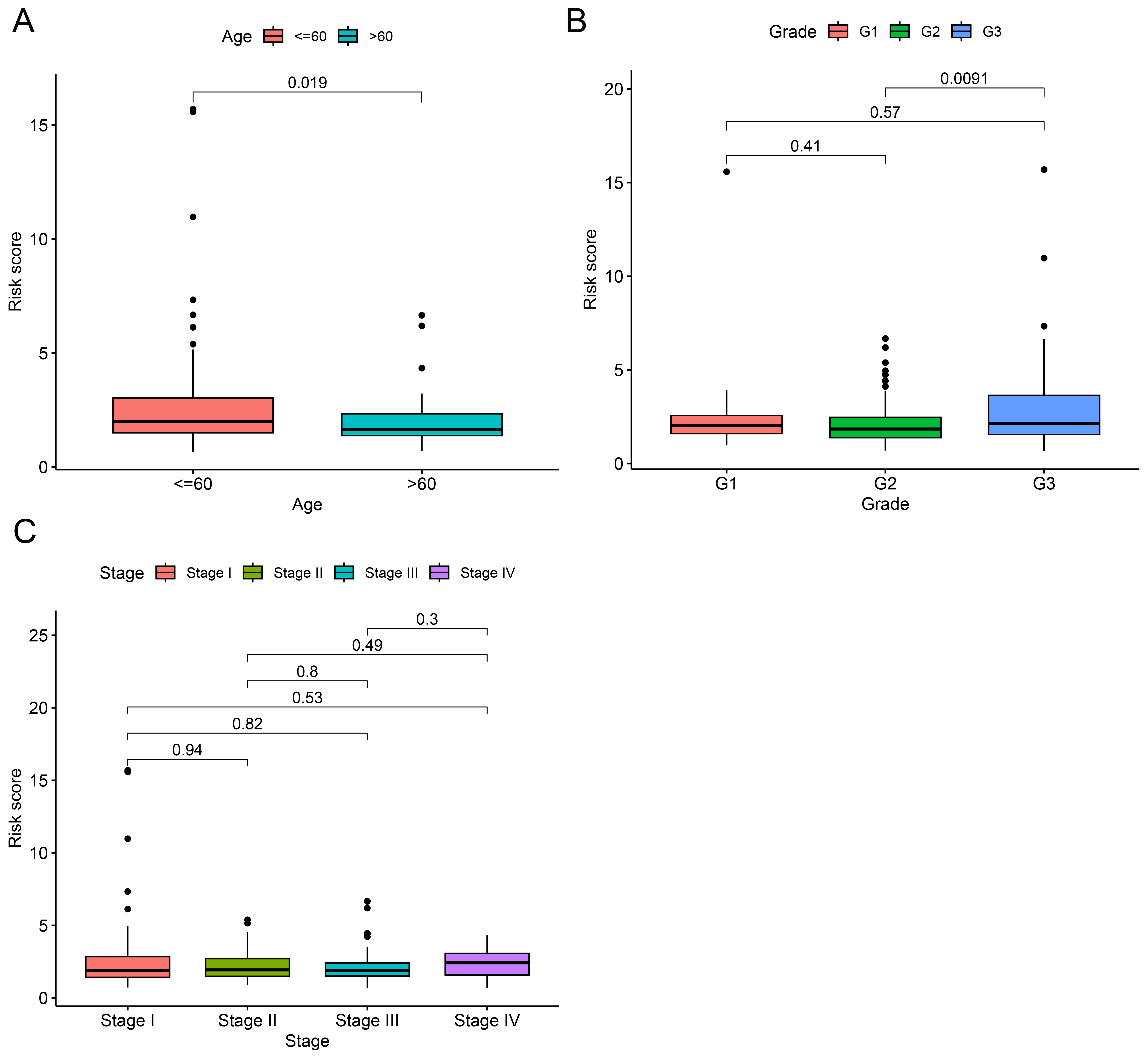

Supplement: Supplementary Figure 4 — Clinical correlation analysis. (A) Association of risk scores with age; (B) Association of risk scores with histologic grade; (C) Association of risk scores with stage. [file Image4.tif]

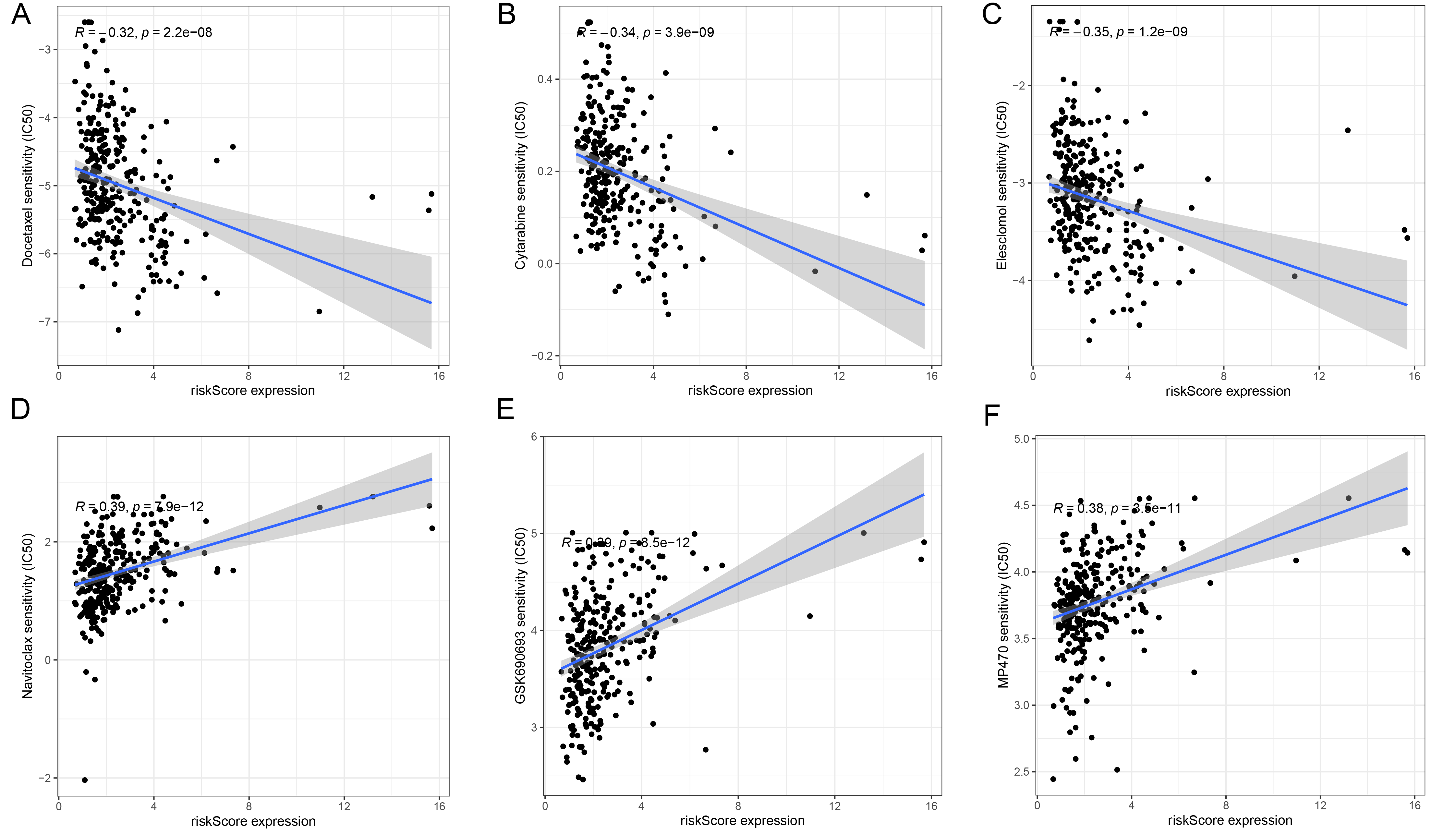

Supplement: Supplementary Figure 5 — Scatter plots depicting the correlation between risk score and drug sensitivity (top 3 genes in positive correlation and negative correlation). [file Image5.tif]

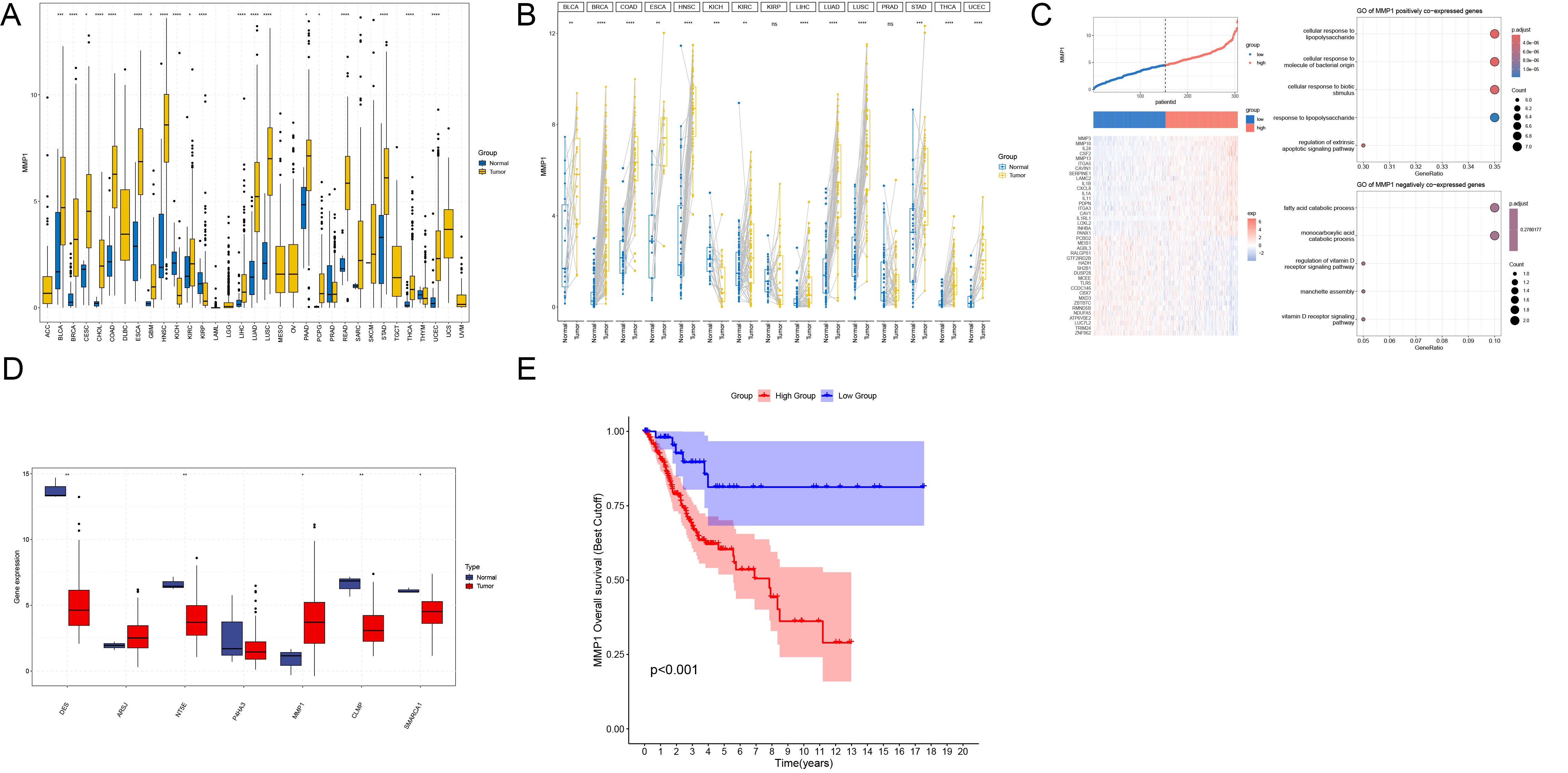

Supplement: Supplementary Figure 6 — Pan-cancer analysis of MMP1, single-gene expression levels, and survival analysis. (A) MMP1 expression in tumor vs. normal tissues (pan-cancer); (B) MMP1 expression in tumor vs. adjacent tissues (pan-cancer); (C) GO enrichment of MMP1 co-expressed genes; (D) Expression levels of core genes in CESC; (E) Single-gene survival analysis of MMP1. [file Image6.tif]

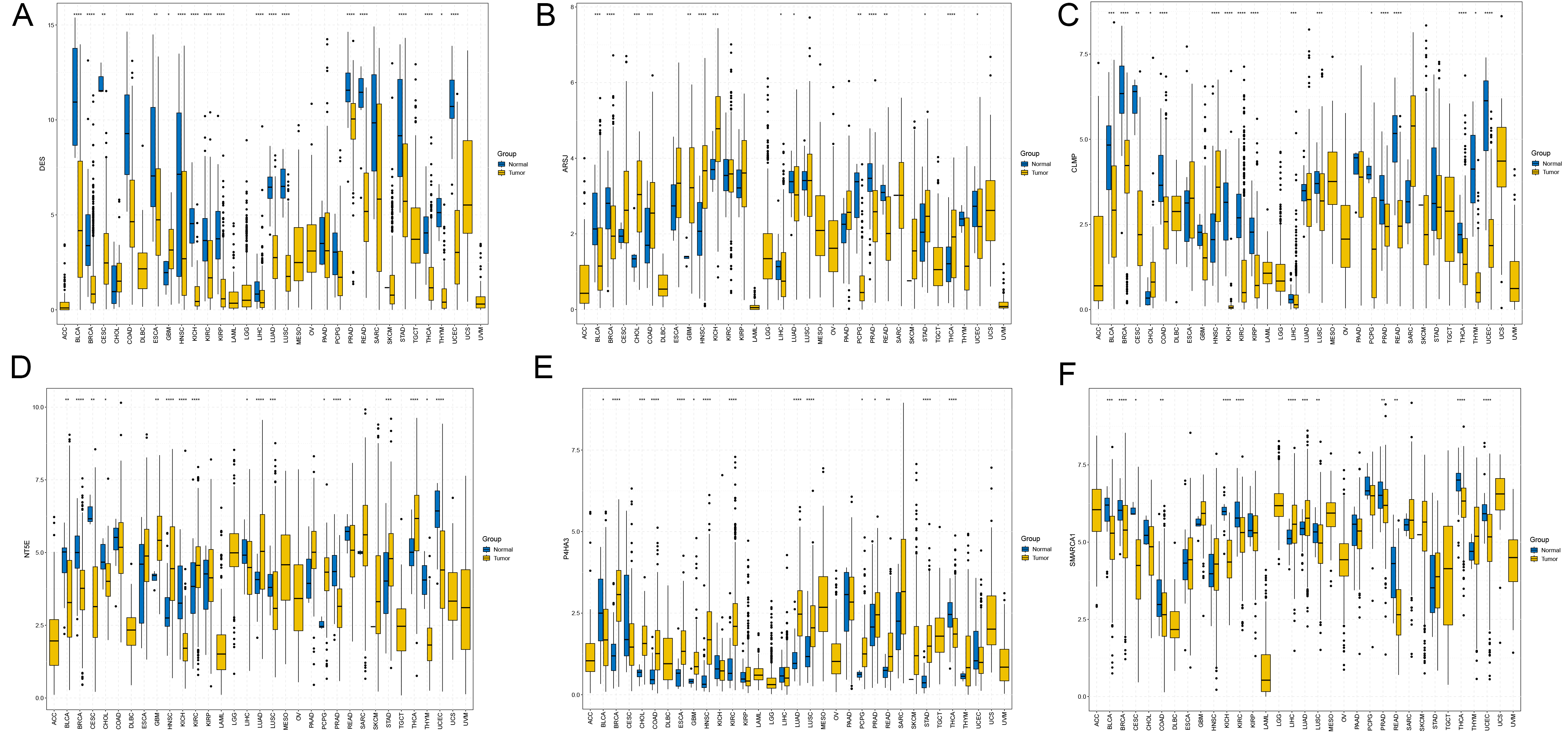

Supplement: Supplementary Figure 7 — Pan-cancer differential expression analysis of core genes between tumor and normal tissues. (A) Expression of DES in pan-cancer tumor vs. normal tissues; (B) Expression of ARSJ in pan-cancer tumor vs. normal tissues; (C) Expression of CLMP in pan-cancer tumor vs. normal tissues; (D) Expression of NT5E in pan-cancer tumor vs. normal tissues; (E) Expression of P4HA3 in pan-cancer tumor vs. normal tissues; (F) Expression of SMARCA1 in pan-cancer tumor vs. normal tissues. [file Image7.tif]

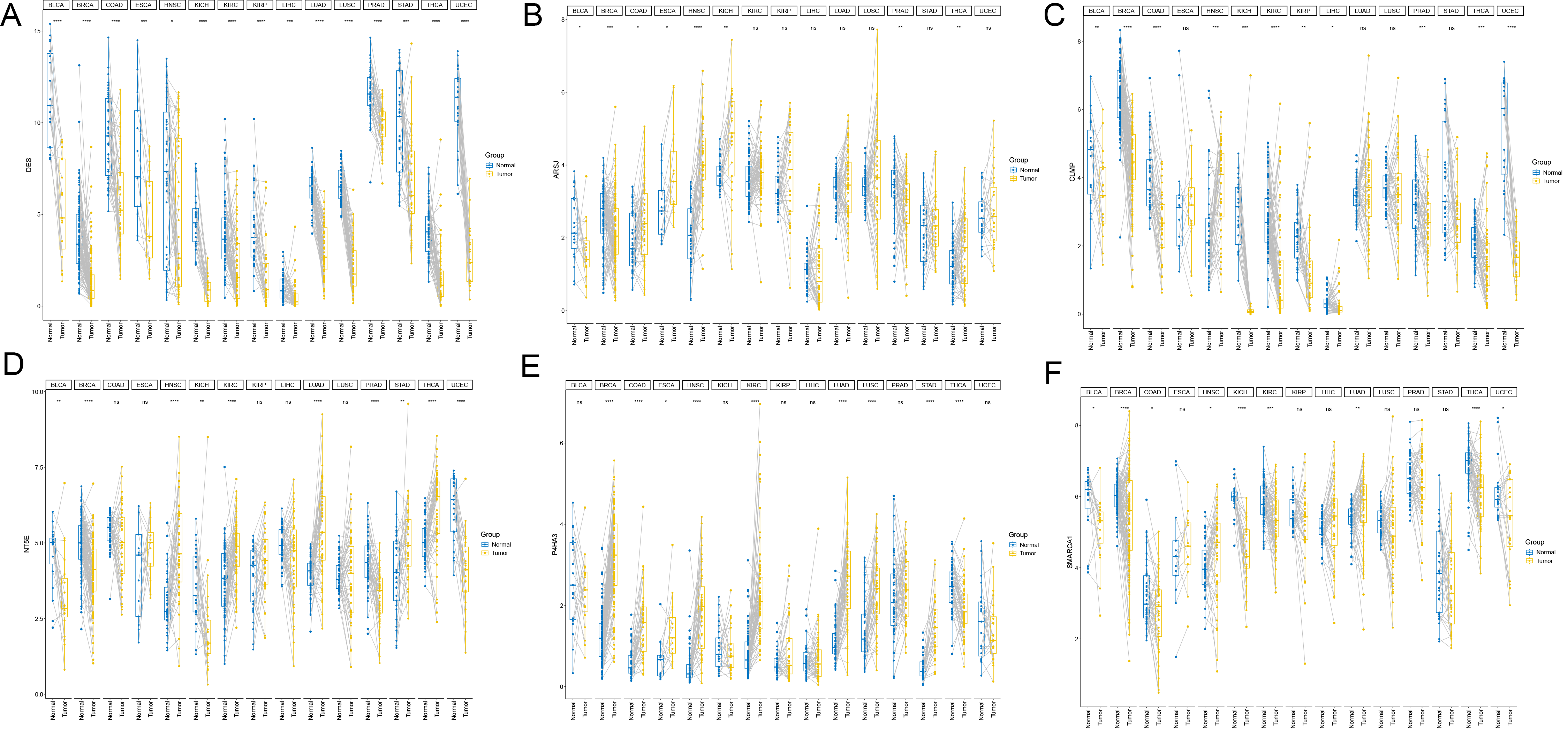

Supplement: Supplementary Figure 8 — Pan-cancer differential expression analysis of core genes between tumor and adjacent tissues. (A) Expression of DES in pan-cancer tumor vs. tumor-adjacent tissues; (B) Expression of ARSJ in pan-cancer tumor vs. tumor-adjacent tissues; (C) Expression of CLMP in pan-cancer tumor vs. tumor-adjacent tissues; (D) Expression of NT5E in pan-cancer tumor vs. tumor-adjacent tissues; (E) Expression of P4HA3 in pan-cancer tumor vs. tumor-adjacent tissues; (F) Expression of SMARCA1 in pan-cancer tumor vs. tumor-adjacent tissues. [file Image8.tif]

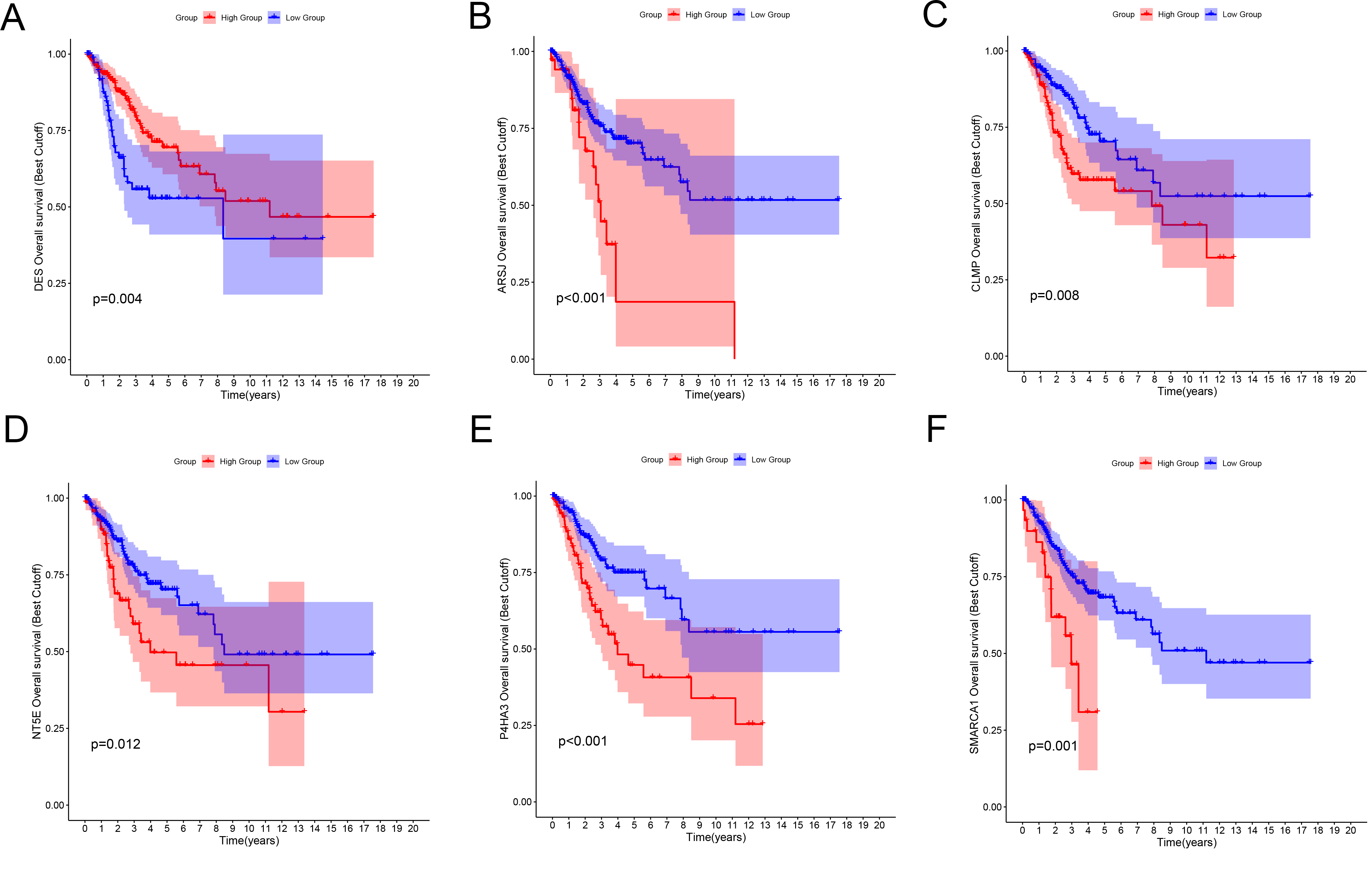

Supplement: Supplementary Figure 9 — Single-gene survival analysis of core genes; (A) DES; (B) ARSJ; (C) CLMP; (D) NT5E; (E) P4HA3; (F) SMARCA1. [file Image9.tif]

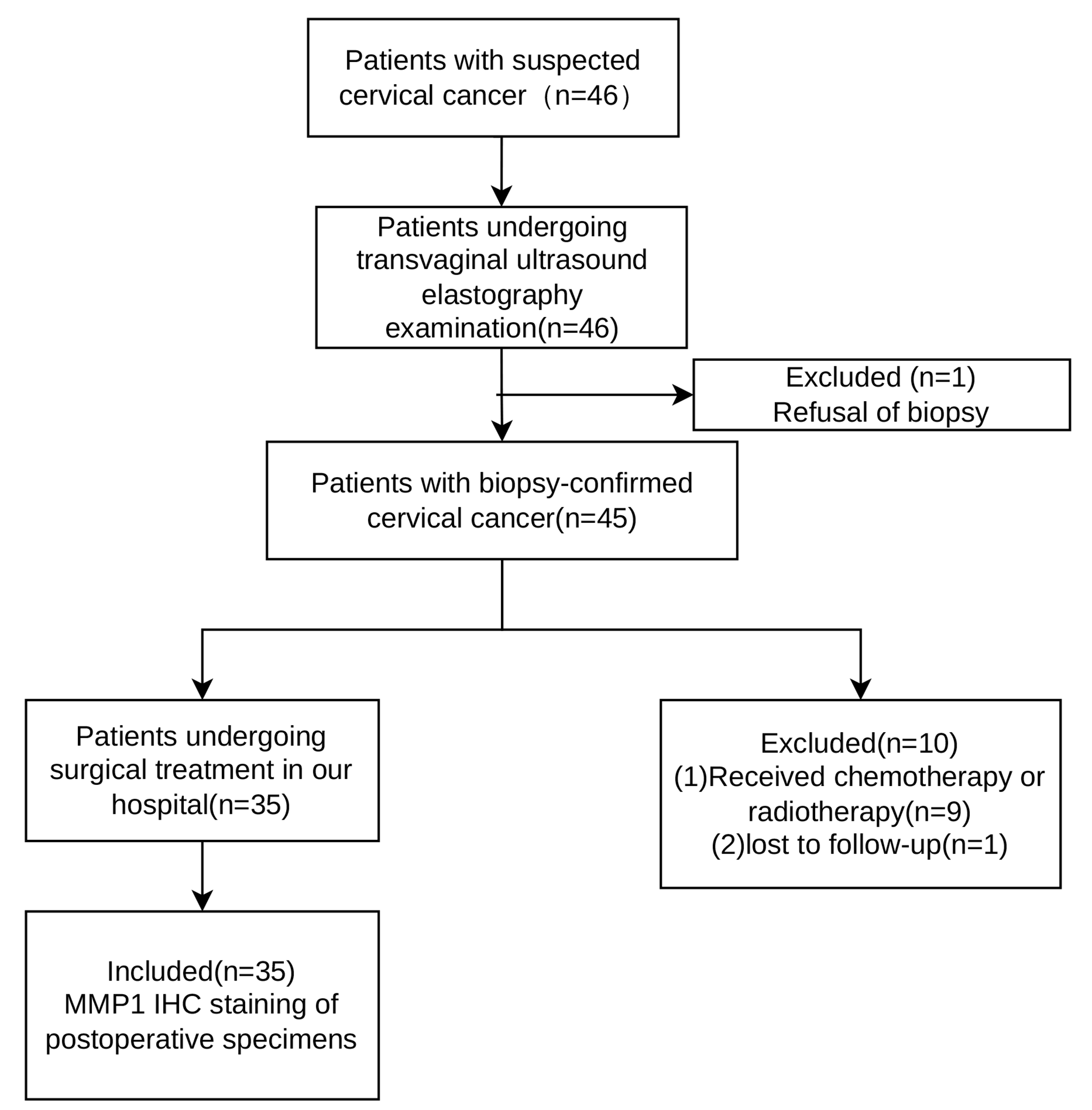

Supplement: Supplementary Figure 10 — Flowchart of the clinical case screening process. [file Image10.tif]
