# Supplementary material for: Preliminary construction and validation of a prognostic prediction model for cervical cancer based on tumor mechanics-related genes
Source: Front Oncol. 2026 Jun 3;16:1841456. doi: 10.3389/fonc.2026.1841456 (PMC13272372; doi:10.3389/fonc.2026.1841456)
Supplement: Supplementary file 11 [file Table1.docx]

Table S1 Primitive cancer mechanics gene set (n=79)

| **Gene symbol** | | | | | |
| --- | --- | --- | --- | --- | --- |
| CGAS | MAPK9 | LOXL4 | MMP8 | YBX1 | DDR2 |
| IRF3 | MAPK10 | LOXL3 | ACTA2 | G3BP2 | SNAIL1 |
| WWTR1 | MAPK11 | LOXL1 | ANLN | CXCR4 | FMN1 |
| YAP1 | MAPK12 | CCN2 | DIAPH3 | CXCL12 | KIF20A |
| EMD | MAPK13 | ITGAV | MYL9 | UBTD1 |  |
| ALOX5 | MAPK14 | ITGB3 | RASSF1A | RAC1 |  |
| PLA2G4A | MAPK15 | ITGB6 | P4HA2 | ENAH |  |
| PTK2 | TWIST1 | ITGA11 | DAB2 | BMP4 |  |
| SRC | TWIST2 | ITGB1 | DOCK1 | FSCN1 |  |
| MAPK1 | MRTFA | ITGB4 | PDGFRA | NTN4 |  |
| MAPK3 | MRTFB | PIEZO1 | VASN | TGFB1 |  |
| MAPK4 | RHOA | MMP9 | PPAP2B | TNC |  |
| MAPK6 | ROCK1 | MMP2 | LPAR1 | BCL9L |  |
| MAPK7 | ROCK2 | MMP14 | LYN | PRKCB |  |
| MAPK8 | LOXL2 | MMP1 | EPHA2 | HIF1A |  |
