# Supplementary material for: Preliminary construction and validation of a prognostic prediction model for cervical cancer based on tumor mechanics-related genes
Source: Front Oncol. 2026 Jun 3;16:1841456. doi: 10.3389/fonc.2026.1841456 (PMC13272372; doi:10.3389/fonc.2026.1841456)
Supplement: Supplementary file 12 [file Table2.docx]

Table S2 Clinical information of samples and sequencing platform in TCGA and GEO.

| Clinical features | TCGA-CESC | GSE 44001 |
| --- | --- | --- |
| OS |  |  |
| 0 | 220 | 262 |
| 1 | 71 | 38 |
| Stage |  |  |
| I | 159 |  |
| II | 64 |  |
| III | 41 |  |
| IV | 21 |  |
| X | 6 |  |
| T stage |  |  |
| T1 | 137 |  |
| T2 | 67 |  |
| T3 | 16 |  |
| T4 | 10 |  |
| TX | 61 |  |
| N stage |  |  |
| N0 | 128 |  |
| N1 | 55 |  |
| NX | 108 |  |
| M stage |  |  |
| M0 | 107 |  |
| M1 | 10 |  |
| MX | 174 |  |
| Grade |  |  |
| G1 | 18 |  |
| G2 | 129 |  |
| G3 | 116 |  |
| G4 | 1 |  |
| GX | 27 |  |
| Sequencing platform | Illumina HiSeq | Illumina HumanHT-12 WG-DASL V4.0 R2 expression beadchip |
